# In-depth clinico-pathological examination of RNA foci in a large cohort of *C9ORF72* expansion carriers

**DOI:** 10.1007/s00401-017-1725-7

**Published:** 2017-05-15

**Authors:** Mariely DeJesus-Hernandez, NiCole A. Finch, Xue Wang, Tania F. Gendron, Kevin F. Bieniek, Michael G. Heckman, Aliaksei Vasilevich, Melissa E. Murray, Linda Rousseau, Rachael Weesner, Anthony Lucido, Meeia Parsons, Jeannie Chew, Keith A. Josephs, Joseph E. Parisi, David S. Knopman, Ronald C. Petersen, Bradley F. Boeve, Neill R. Graff-Radford, Jan de Boer, Yan W. Asmann, Leonard Petrucelli, Kevin B. Boylan, Dennis W. Dickson, Marka van Blitterswijk, Rosa Rademakers

**Affiliations:** 1 Department of Neuroscience, Mayo Clinic, 4500 San Pablo Road, Jacksonville, FL 32224 USA; 2 Department of Health Sciences Research, Mayo Clinic, 4500 San Pablo Road, Jacksonville, FL 32224 USA; 3 Division of Biomedical Statistics and Informatics, Mayo Clinic, 4500 San Pablo Road, Jacksonville, FL 32224 USA; 4 MERLN Institute for Technology-inspired Regenerative Medicine, Maastricht University, Universiteitssingel 40, 6229 ER Maastricht, The Netherlands; 5 Department of Neurology, Mayo Clinic, 200 1st St SW, Rochester, MN 55905 USA; 6 Department of Neurology, Mayo Clinic, 4500 San Pablo Road, Jacksonville, FL 32224 USA

**Keywords:** C9ORF72, Frontotemporal dementia, Frontotemporal lobar degeneration, Motor neuron disease, Amyotrophic lateral sclerosis, RNA foci

## Abstract

A growing body of evidence suggests that a loss of chromosome 9 open reading frame 72 (*C9ORF72*) expression, formation of dipeptide-repeat proteins, and generation of RNA foci contribute to disease pathogenesis in amyotrophic lateral sclerosis and frontotemporal dementia. Although the levels of *C9ORF72* transcripts and dipeptide-repeat proteins have already been examined thoroughly, much remains unknown about the role of RNA foci in *C9ORF72*-linked diseases. As such, we performed a comprehensive RNA foci study in an extensive pathological cohort of *C9ORF72* expansion carriers (*n* = 63). We evaluated two brain regions using a newly developed computer-automated pipeline allowing recognition of cell nuclei and RNA foci (sense and antisense) supplemented by manual counting. In the frontal cortex, the percentage of cells with sense or antisense RNA foci was 26 or 12%, respectively. In the cerebellum, 23% of granule cells contained sense RNA foci and 1% antisense RNA foci. Interestingly, the highest percentage of cells with RNA foci was observed in cerebellar Purkinje cells (~70%). In general, more cells contained sense RNA foci than antisense RNA foci; however, when antisense RNA foci were present, they were usually more abundant. We also observed that an increase in the percentage of cells with antisense RNA foci was associated with a delayed age at onset in the frontal cortex (*r* = 0.43, *p* = 0.003), whereas no other associations with clinico-pathological features were seen. Importantly, our large-scale study is the first to provide conclusive evidence that RNA foci are not the determining factor of the clinico-pathological variability observed in *C9ORF72* expansion carriers and it emphasizes that the distribution of RNA foci does not follow the pattern of neurodegeneration, stressing the complex interplay between different aspects of *C9ORF72*-related diseases.

**Electronic supplementary material:**

The online version of this article (doi:10.1007/s00401-017-1725-7) contains supplementary material, which is available to authorized users.

## Introduction

Three mechanisms by which a repeat expansion in chromosome 9 open reading frame 72 (*C9ORF72*) might be causative of amyotrophic lateral sclerosis (ALS) and frontotemporal dementia (FTD) [11, 31] are a loss of *C9ORF72* expression [11], the formation of dipeptide-repeat proteins aberrantly translated from the repeat [2, 27], and the generation of RNA foci containing flawed RNA transcripts [11]. The contribution of each of those mechanisms to disease pathogenesis, however, remains largely unknown. While studies focusing on the levels of *C9ORF72* transcripts and dipeptide-repeat proteins did not fully explain the clinical and pathological variability observed in *C9ORF72* expansion carriers [15, 39], we now seek to elucidate the role that RNA foci play in *C9ORF72*-linked diseases.

The first report revealing RNA foci in patients carrying *C9ORF72* repeat expansions demonstrated that they are present in approximately 25% of cells in the frontal cortex and spinal cord, and it has been hypothesized that they may sequester RNA-binding proteins, potentially disrupting mRNA splicing [11]. In fact, RNA foci have been found to co-localize with various proteins [9, 10, 12, 20, 32, 33], such as heterogeneous nuclear ribonucleoproteins (hnRNPs), purine-rich element binding (Pur)-alpha, adenosine deaminase RNA specific B2 (ADARB2), and Aly/REF export factor (ALYREF). It has also been shown that RNA foci can contain both sense and antisense transcripts [14, 43], which suggests that bidirectional transcription occurs.

Additionally, RNA foci have been studied in several model systems, including lymphoblast cell lines, fibroblast cell lines, induced pluripotent stem cells (iPSCs) as well as iPSC-derived motor neurons, oligodendrocytes, and skeletal muscle [1, 8, 12, 19, 21, 24, 33, 35, 42, 43]. Other models, such as zebrafish, flies, and mice, have also been generated [7, 17, 20, 23, 28–30, 37] and revealed RNA foci in numerous central nervous system tissues [7]. An in-depth study focusing on a large cohort of patients harboring *C9ORF72* repeat expansions, however, has not yet been reported. Therefore, it is unclear whether RNA foci associate with clinical or pathological features of ALS and/or FTD. As such, we have performed an extensive clinico-pathological study examining sense and antisense foci in a cohort of *C9ORF72* expansion carriers obtained from the Mayo Clinic Florida Brain Bank (*n* = 63).

## Materials and methods

### Subjects

We selected all *C9ORF72* expansion carriers from the Mayo Clinic Florida Brain Bank, for whom formalin-fixed paraffin-embedded (FFPE) sections from the frontal cortex and/or cerebellum could be acquired (*n* = 63; Table 1). Of these expansion carriers, 56% was male (*n* = 35), their median age at death was 66.8 years, and their median survival after onset was 4.9 years. They had received a pathological diagnosis of predominant frontotemporal lobar degeneration (FTLD; *n* = 26), predominant motor neuron disease (MND; *n* = 18), or mixed pathology (FTLD/MND; *n* = 16). Additionally, we included a patient with a primary pathological diagnosis of Alzheimer’s disease (AD; *n* = 1) [3] and patients clinico-pathologically diagnosed with depressive pseudodementia (*n* = 2) [4]. For the majority of samples, *C9ORF72* transcript levels, dipeptide-repeat protein levels, and expansion sizes were previously determined [15, 38, 39].

**Table 1:** Subject characteristics

Variable	Overall cohort (*n* = 63)
Gender (male)	35 (56%)
Age at onset (years)	62.1 (55.0–67.3)
Age at death (years)	66.8 (60.2–72.8)
Survival after onset (years)	4.9 (2.8–8.0)
Diagnosis	
FTLD	26 (41%)
FTLD/MND	16 (25%)
MND	18 (29%)
Other	3 (5%)
Available frontal cortex	
All cells sense	49 (78%)
All cells antisense	50 (79%)
Neurons sense	49 (78%)
Neurons antisense	50 (79%)
Available cerebellum	
Granule cells sense	51 (81%)
Granule cells antisense	59 (94%)
Purkinje cells sense	58 (92%)
Purkinje cells antisense	62 (98%)

Data are sample median (IQR) or number (%). Information was obtained for patients with expansions in *C9ORF72*. This study was performed in the frontal cortex and cerebellum. Information was unavailable regarding age at onset (*n* = 5), age at death (*n* = 1), and survival after onset (*n* = 5)

### RNA fluorescent in situ hybridization (FISH) and immunofluorescence staining

Sections were cut at a thickness of 5 μm and mounted onto glass slides. RNA FISH was performed using 5′ TYE-563-labeled locked nucleic acid (LNA) probes complementary to either sense RNA foci containing GGGGCC-repeats (Exiqon; batch 616667) or to antisense RNA foci with GGCCCC-repeats (Exiqon; batch 619229). Slides were deparaffinized, rehydrated, and washed, followed by an antigen retrieval treatment step with citrate buffer (0.1 M) for 20 min at 37 °C. Hereafter, they were permeabilized in 20% ice-cold acetic acid for 90 s. Slides were then dehydrated with 70, 90, and 100% ethanol. After an air drying step, they were pre-hybridized with 50% formamide/2X saline-sodium citrate (SSC) buffer for 1 h at 80 °C. To allow hybridization to our probes, they were diluted to 0.8 ng/μl in hybridization buffer [10% dextran sulfate, 50% formamide, 20 ng/μl bovine serum albumin (BSA), 25 mM tRNA, 25 nM ethylenediaminetetraacetic acid (EDTA), 2X SSC, and 25 mM sodium phosphate buffer] and denatured for 5 min at 80 °C. Hybridization was performed in a humidified light protected chamber for 20 h at 80 °C. Next, coverslips were removed and slides were washed: once in 2X SSC, three times in 50% formamide/0.5X SSC for 5 min at 80 °C, and three times in 1X SSC for 5 min at room temperature. Finally, slides were treated with 0.2% Sudan Black B in 70% ethanol for 2 min and washed three times with 1X phosphate-buffered saline (PBS), before they were coverslipped and mounted using ProLong^®^ Gold anti-fade reagent with DAPI (Invitrogen).

To allow assessment of specific cell types, immunofluorescence staining was performed after RNA FISH (Online Resource Figs. 1 and 2). In brief, slides were blocked in 10% fetal bovine serum (FBS) buffer for 30 min at room temperature, washed three times in 1X PBS, and incubated with a primary antibody overnight at 4 °C. Subsequently, they were incubated with an Alexa Fluor 488-conjugated secondary antibody (1:1000) for 1 h at room temperature, washed with 1X PBS, and treated with 0.2% Sudan Black B in 70% ethanol for 2 min. They were then washed three times in 1X PBS, before they were coverslipped and mounted using ProLong^®^ Gold anti-fade reagent with DAPI. The following primary antibodies were used: mouse monoclonal microtubule-associated protein 2 antibody (MAP2; Sigma; 1:1000) to recognize neuronal cells, rabbit polyclonal glial fibrillary acidic protein antibody (GFAP; Abcam; 1:500) to recognize astrocytes, rabbit polyclonal Iba1 antibody (Wako; 1:2000) to recognize microglia, rabbit monoclonal carbonic anhydrase II antibody (CAII; Abcam; 1:1000) to recognize oligodendrocytes, and rabbit polyclonal calbindin D-28K antibody (1:500; Swant) to recognize Purkinje cells.

Stained slides were imaged using a Zeiss Axio Imager Z1 microscope with a 63X oil objective. For the frontal cortex, we captured the middle frontal gyrus at the level of the external pyramidal layer (III) through the internal granular layer (IV), the internal pyramidal layer (V), and the multiform layer (VI), adjacent to the white matter. The cerebellar vermis was also imaged, focusing on the Purkinje cell layer that separates the granular and molecular layers. A series of Z-stack images was acquired with a distance of 0.24 μm between individual planes to span the entire thickness of cells. Brightness and contrast were adjusted to reduce the background signal prior to image processing using our computer-automated pipelines.

### Recognition of RNA foci and cells

**Fig. 1:**
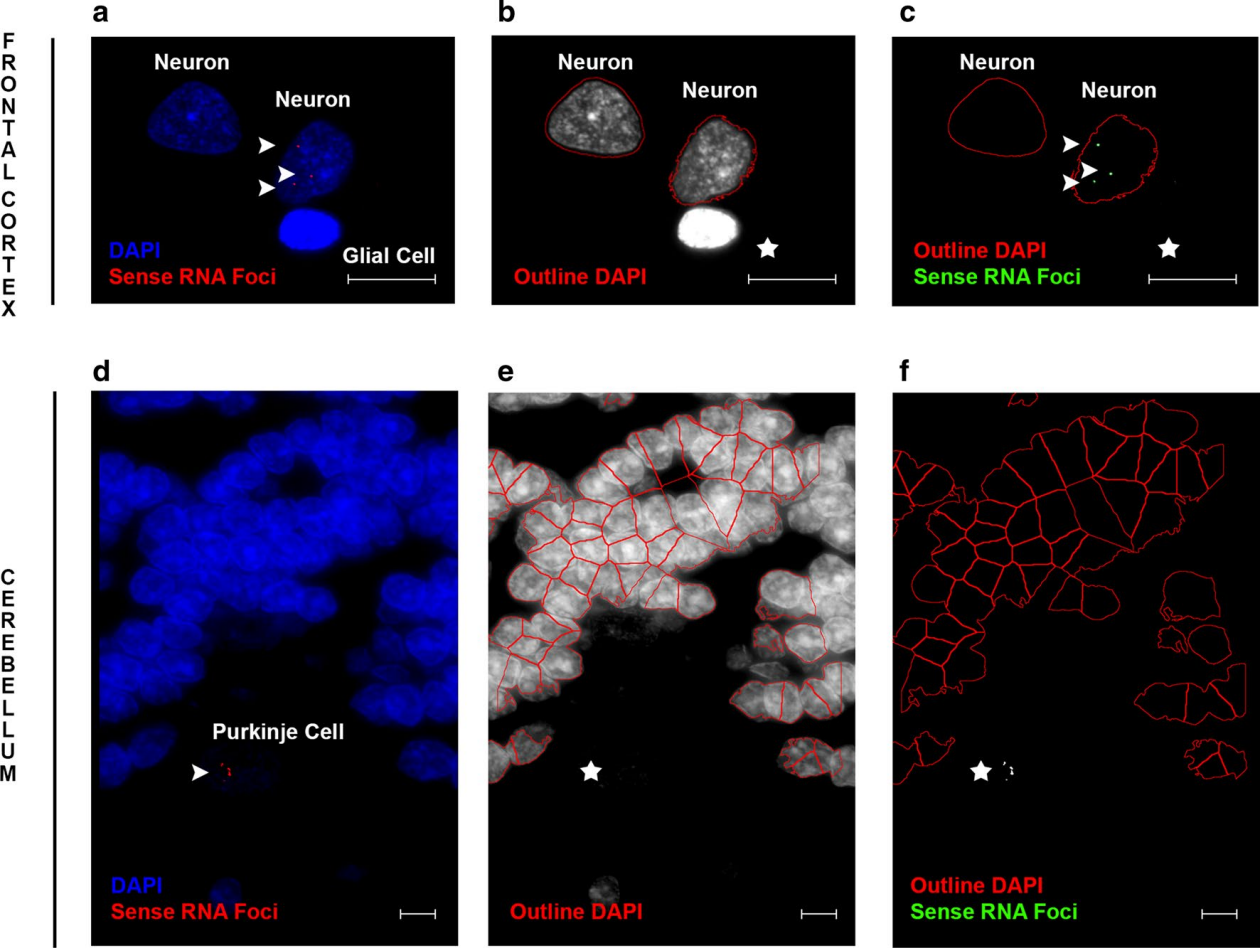
Examples of computer-automated recognition of specific cell types. Images are displayed to demonstrate the recognition of specific cell types in either the frontal cortex (*top panel*) or cerebellum (*bottom panel*). Cell nuclei (*blue* DAPI) and RNA foci (*red* RNA foci) are shown for images taken in the frontal cortex (**a**) and cerebellum (**d**). Our computer-automated pipelines are able to recognize neuronal nuclei in the frontal cortex (*red outline* nucleus), while ignoring glial nuclei (*star*; **b**). Additionally, they recognize cerebellar granule cells, without recognizing Purkinje cells that stain poorly with DAPI (*star*; **e**). RNA foci are shown in neuronal nuclei (*green* RNA foci; **c**); however, they are not being recognized in cerebellar Purkinje cells (*star*; **f**). RNA foci that might be difficult to see are highlighted by *arrowheads*. *Scale bars* 5 µm (*top panel*) or 2 µm (*bottom panel*)

For the frontal cortex, multiple Z-stack images were obtained from each individual; on average 24 for sense RNA foci and 21 for antisense RNA foci. The Cy3 channel contained information on RNA foci (either sense or antisense) and the DAPI channel on cell nuclei. CellProfiler 2.2.0 [6] was utilized to merge images and to perform a computer-automated recognition of RNA foci and cell nuclei (our pipelines are available upon request). RNA foci were defined as bright speckles that were present in cell nuclei and whose diameter ranged from 1 to 11 pixels; larger objects with a similar intensity were considered too big to represent RNA foci and were masked from downstream analyses. Since we were particularly interested in neuronal cells in the frontal cortex, a supervised random forest classifier was applied, ilastik 0.5.12 [34]; 10 randomly selected images were used to train ilastik. This information was then fed into CellProfiler, which identified putative neuronal cells given their intensity, edges, texture, diameter, and area (Fig. 1). In addition, we also developed an automated pipeline that did not differentiate between neuronal cells and glial cells, but rather recognized all cell nuclei, taking their intensity, diameter, area, and shape into consideration (Online Resource Figs. 3 and 4).

For the cerebellum, each expansion carrier had an average of 10 Z-stack images to assess sense RNA foci and 17 for antisense RNA foci. Similar to the frontal cortex, CellProfiler was used to merge images and to count the number of RNA foci and cell nuclei. RNA foci were between 1 and 10 pixels in size and had to be located within a nucleus. Nuclei of granule cells were recognized based on their intensity and shape, using a propagation method to divide clumped cells (Fig. 1; Online Resource Figs. 3 and 5).

In addition to using our computer-automated pipelines, a subset of Z-stack images was counted manually by two independent investigators. Specifically, RNA foci in Purkinje cells were counted manually using all cerebellar images, because Purkinje cell nuclei stained poorly with DAPI (Fig. 1), hampering their detection using our computer-automated pipelines. Nuclei and RNA foci of 20 randomly selected images were also counted manually for each brain region, allowing validation of our computer-automated pipelines.

Using aforementioned methods in the frontal cortex, we assessed sense RNA foci in, on average, 358 cell nuclei per individual, including roughly 260 neuronal nuclei. For antisense RNA foci, we evaluated approximately 437 cell nuclei per individual, of which 230 were designated as neurons. In the cerebellum, an average of 1187 granule cell nuclei was counted per individual, when examining sense RNA foci; an average of 2021 granule cell nuclei was assessed for antisense RNA foci. Per individual, an average of 11 and 21 Purkinje cell nuclei was examined, stained for sense or antisense RNA foci, respectively.

### Statistical analysis

Five different RNA foci outcomes were measured: (1) the percentage of cells with RNA foci, (2) the mean number of RNA foci in all cells, (3) the mean number of RNA foci in cells containing foci, (4) the maximum number of RNA foci, and (5) the total number of RNA foci. For each of these measurements, we performed two analyses in the frontal cortex (all cells and enriched for neuronal cells) and cerebellum (granule cells and Purkinje cells). We summarized data with median and interquartile range (IQR; Table 2).

**Table 2:** RNA foci measurements in two brain regions (overall cohort)

Tissue	Variable				
Frontal cortex		All cells sense	All cells antisense	Neurons sense	Neurons antisense
	Percentage of cells	0.26 (0.20–0.31)	0.12 (0.06–0.16)	0.32 (0.23–0.37)	0.16 (0.07–0.24)
	Mean number of foci (all)	0.61 (0.38–0.79)	0.50 (0.15–0.84)	0.73 (0.50–0.97)	0.82 (0.23–1.34)
	Mean number of foci (pos)	2.12 (1.86–2.52)	3.89 (2.89–5.47)	2.25 (1.96–2.66)	4.34 (3.02–5.66)
	Maximum number of foci	11 (8–13)	22 (14–38)	10 (8–13)	21 (13–37)
	Total number of foci	207 (149–248)	182 (67–348)	194 (131–230)	147 (47–299)
Cerebellum		Granule cells sense	Granule cells antisense	Purkinje cells sense	Purkinje cells antisense
	Percentage of cells	0.23 (0.16–0.29)	0.01 (0.009–0.02)	0.70 (0.58–0.80)	0.74 (0.58–0.83)
	Mean number of foci (all)	0.31 (0.21–0.40)	0.02 (0.01–0.03)	3.55 (2.29–5.75)	7.83 (4.42–12.11)
	Mean number of foci (pos)	1.38 (1.28–1.44)	1.35 (1.23–1.47)	5.50 (3.62–7.60)	11.54 (6.10–15.07)
	Maximum number of foci	6 (4–7)	4 (3–5)	14 (8–22)	39 (23–50)
	Total number of foci	342 (254–485)	38 (22–65)	38 (25–62)	172 (82–268)

Data are sample median (IQR). Sense and antisense RNA foci measurements are shown in the frontal cortex for all cells and neurons, while they are shown for granule cells and Purkinje cells in the cerebellum. Five statistical RNA foci measurement are investigated: the percentage of cells with RNA foci, the mean number of RNA foci in all cells, the mean number of RNA foci in foci-positive cells, the maximum number of RNA foci, and the total number of RNA foci

For the subset of patients for whom sense and antisense RNA foci were determined in both brain regions (*n* = 41), each of these five measurements was compared between cell types (all cells versus neuronal cells; granule cells versus Purkinje cells), tissues (frontal cortex versus cerebellum), and RNA foci (sense versus antisense), using a paired Wilcoxon signed rank test.

In our overall cohort, associations between the different RNA foci measurements were investigated using a Spearman’s test of correlation [estimating Spearman’s correlation coefficient *r* and 95% confidence interval (CI)]. We then evaluated whether each RNA foci measurement was associated with clinical or pathological features of the disease, including age at onset, age at death, repeat length, total *C9ORF72* transcripts, variant 1 transcripts, variant 2 transcripts, variant 3 transcripts, intron 1a containing transcripts, intron 1b containing transcripts, dipeptide-repeat proteins [poly(GP) and poly(GA)], gender, disease subgroups, and survival after onset (using the median as cutoff). A Spearman’s test of correlation, a Wilcoxon rank sum test, or a Kruskal–Wallis rank sum test were used, as appropriate for the nature of the given clinical or pathological feature. For survival after onset analysis, a Cox proportional hazards regression model was used [estimating hazard ratios (HRs) and 95% CIs; using deaths of any cause as endpoint], adjusting for age at onset and disease subgroup. A Bonferroni correction was utilized to adjust for multiple testing, separately for each group of similar statistical tests. All statistical tests were two-sided and were performed using R Statistical Software (R Foundation for Statistical Computing, version 3.3.2).

## Results

### Strong correlation between computer-automated pipelines and manual counts

We examined RNA foci in patients carrying a repeat expansion in *C9ORF72* (*n* = 63), using computer-automated pipelines combined with manual counting. RNA FISH coupled with immunofluorescence staining revealed neuronal cells as well as glial cells (e.g., astrocytes, microglia, and oligodendrocytes; Online Resource Figs. 1 and 2); RNA foci were often seen in neuronal cells, but were rare in glial cells, aligning with previous studies [19, 26]. In the frontal cortex, we optimized our pipelines to recognize all cell nuclei or to enrich for neuronal nuclei (Fig. 1; Online Resource Fig. 4). Our pipelines also recognized granule cell nuclei in the cerebellum; however, Purkinje cell nuclei stained poorly with DAPI (Fig. 1; Online Resource Fig. 5), and consequently, they were counted manually by two independent investigators.

After optimizing our pipelines, 20 randomly selected Z-stack images were used for validation, revealing a strong correlation between our computer-automated pipelines and manual counts, both in the frontal cortex (all cells: *r* = 0.77, *p* = 7.30e-05; neurons: *r* = 0.82, *p* = 9.34e-06; Online Resource Fig. 6) and cerebellum (granule cells: *r* = 0.95, *p* = 1.13e-10; Online Resource Fig. 7). We also noticed a strong correlation between manual counting by two independent investigators (*r* ≥ 0.78, *p* ≤ 4.78e-05; Online Resource Figs. 6 and 7).

### Frequent RNA foci in brain regions studied

**Fig. 2:**
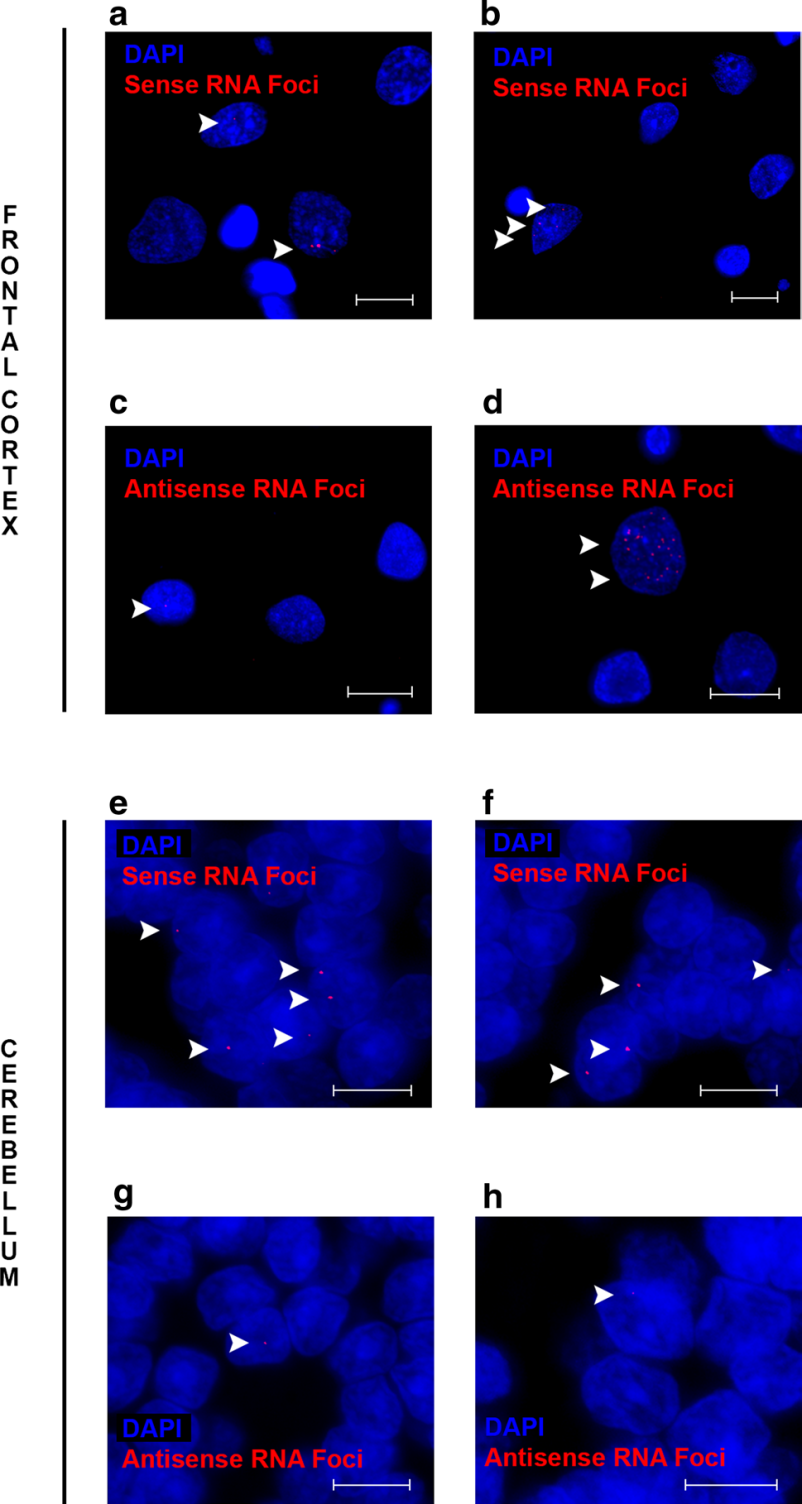
Examples of frequently observed RNA foci patterns. Representative examples are shown of images with cell nuclei (*blue* DAPI) and RNA foci (*red* RNA foci) to demonstrate common patterns. In the frontal cortex, sense RNA foci are generally absent or a small number is seen (**a**, **b**). Antisense RNA foci are less frequently encountered, but if cells contain antisense RNA foci, then their numbers vary from just a few (**c**) to many (**d**). In the cerebellum, granule cells generally contain either no sense RNA foci or a relatively low number (**e**, **f**). Cerebellar antisense RNA foci are rare in granule cells, and if they are present, only one or two are detected (**g**, **h**). RNA foci that might be difficult to see are highlighted by *arrowheads*. *Scale bars* 5 µm (frontal cortex) or 2 µm (cerebellum)

**Fig. 3:**
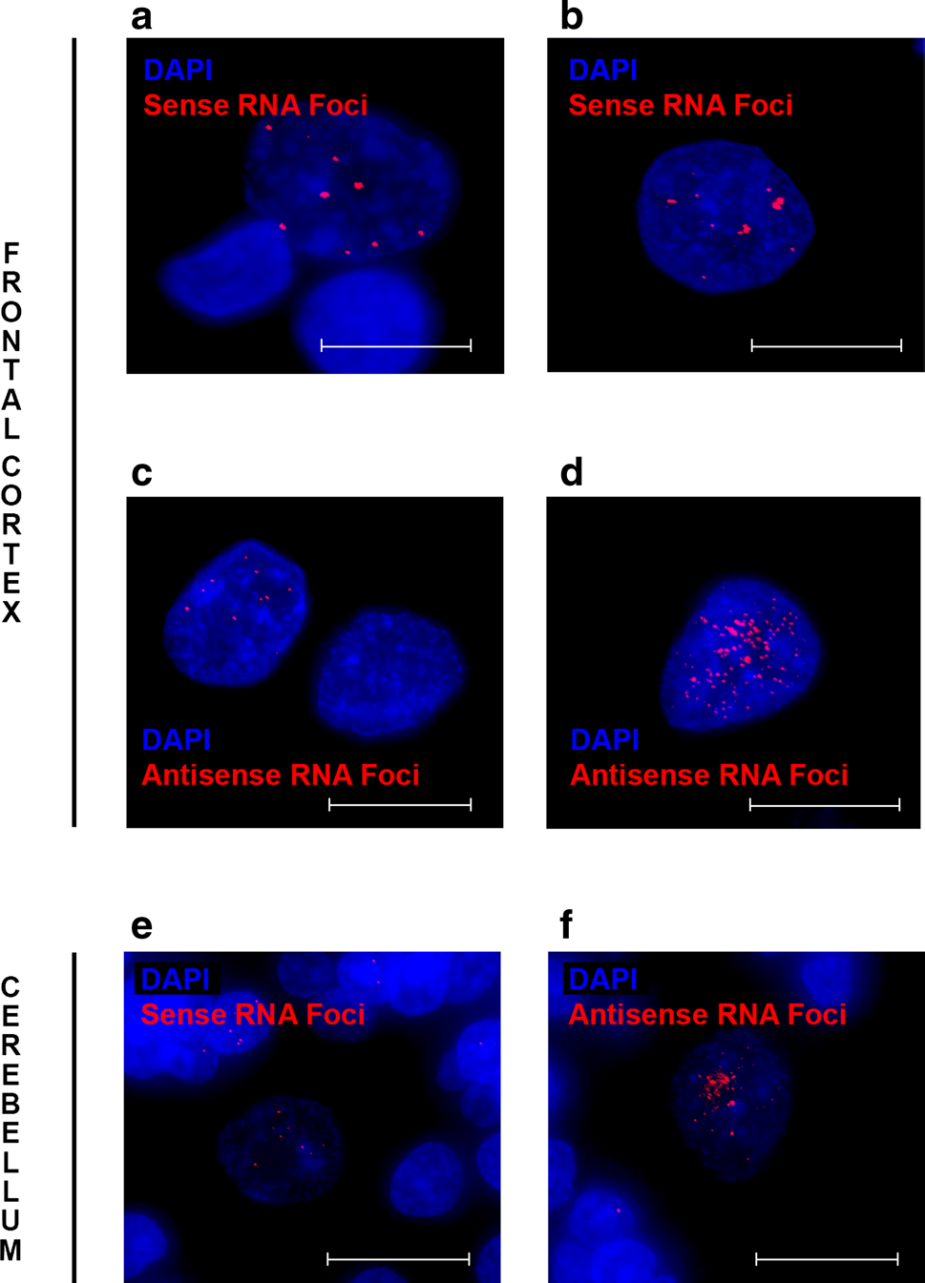
Examples of cells with a large number of RNA foci. Occasionally, cells with abundant RNA foci are observed, both in the frontal cortex (**a**–**d**) and cerebellum (**e**, **f**). Images are shown that display numerous RNA foci (*red* RNA foci) within a single nucleus (*blue* DAPI). Sense RNA foci are included (**a**, **b**, **e**) as well as antisense RNA foci (**c**, **d**, **f**). Please note the faint DAPI signal in Purkinje cells as compared to other cells (**e**, **f**). *Scale bars* 5 µm

In the frontal cortex, the percentage of cells with sense RNA foci was 26% overall and 32% in neurons (Table 2); antisense RNA foci were observed in 12% of cells and 16% of neurons. In the cerebellum, 23% of granule cells contained sense RNA foci and 70% of Purkinje cells. Of the granule cells and Purkinje cells, 1 and 74% harbored antisense RNA foci, respectively. In general, RNA foci appeared to be present in a greater proportion of cells than antisense RNA foci (Fig. 2), with the exception of cerebellar Purkinje cells. A relatively small number of RNA foci (e.g., one, two, or three; Fig. 2) was observed in the majority of RNA foci-positive cells, but occasionally they were much more abundant (Fig. 3), particularly antisense RNA foci.

### Cell type-specific differences in RNA foci pattern

**Fig. 4:**
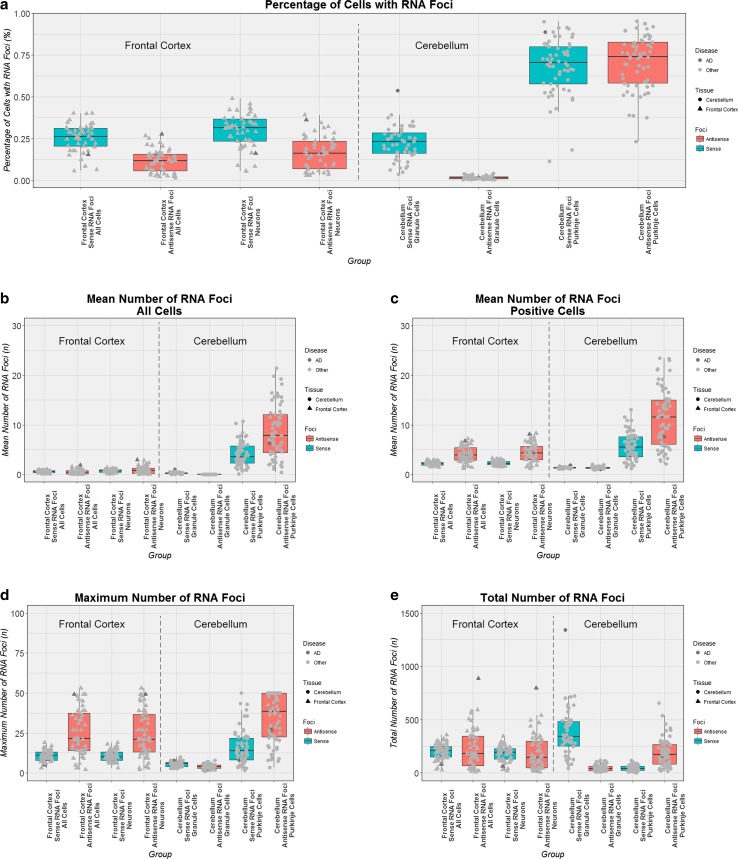
Cell type- and tissue-specific differences in RNA foci measurements. Every *box plot* visualizes a different RNA foci measurement: the percentage of cells with RNA foci (**a**), the mean number of RNA foci in all cells (**b**), the mean number of RNA foci in cells with foci (**c**), the maximum number of RNA foci (**d**), and the total number of RNA foci (**e**). For each *box plot*, the median is represented by a *solid black line*, and each *box* spans the interquartile range (IQR; 25th percentile to 75th percentile). On the *left side* of all plots the frontal cortex is displayed, where each patient is represented by a *solid triangle*. On the *right side* the cerebellum is shown with *solid circles* representing patients. One expansion carrier with a primary pathological diagnosis of Alzheimer’s disease (AD) is highlighted in *dark grey*, whereas other patients are shown in *light grey*. *Boxes* that are *turquoise* denote sense RNA foci, whereas *salmon boxes* denote antisense RNA foci

Next, we examined the subset of patients for whom sense and antisense RNA foci were assessed in both brain regions (*n* = 41) to allow comparisons between cell types, tissue types, and RNA foci types (Fig. 4). In the frontal cortex, the percentage of cells with sense RNA foci was significantly higher when enriching for neurons than in all cells (*p* = 3.39e-08; Online Resource Table 1). Furthermore, the mean number of sense RNA foci was significantly increased in neurons compared to all cells (*p* ≤ 5.47e-08). The maximum number of sense RNA foci, however, was not significantly different between groups (*p* = 0.31). As expected, the total number of RNA foci observed in all cells was significantly greater than in neurons (*p* = 5.37e-08). A similar pattern was seen for antisense RNA foci (Online Resource Table 1).

In the cerebellum, the percentage of cells with sense RNA foci was significantly higher in Purkinje cells compared to granule cells (*p* = 2.52e-08; Online Resource Table 1). Moreover, Purkinje cells contained significantly more sense RNA foci than granule cells (*p* = 2.52e-08). The maximum number of sense RNA foci was also significantly elevated in cerebellar Purkinje cells when comparing them to granule cells (*p* = 1.70e-07). Not surprisingly, considering the low number of Purkinje cells, the total number of sense RNA foci was significantly higher in granule cells than in Purkinje cells (*p* = 2.52e-08). Again, the pattern was comparable for antisense RNA foci, except for the total number of RNA foci that was significantly lower in granule cells compared to Purkinje cells (*p* = 7.53e-08; Online Resource Table 1).

### Tissue-specific differences in RNA foci pattern

When comparing neuronal cells in the frontal cortex to granule cells in the cerebellum, the percentage of cells as well as the mean and maximum number of sense RNA foci was significantly higher in frontal cortex neurons than in cerebellar granule cells (*p* ≤ 3.57e-05; Online Resource Table 2). The total number of sense RNA foci, however, was significantly higher in cerebellar granule cells (*p* = 3.50e-06). Findings were comparable for antisense RNA foci, with the exception of the total number of RNA foci, which was significantly lower in cerebellar granule cells than in frontal cortex neurons (*p* = 4.09e-06; Online Resource Table 2).

We then compared frontal cortex neurons to cerebellar Purkinje cells. The percentage of cells with sense RNA foci, but also the mean and maximum number of sense RNA foci was significantly smaller in frontal cortex neurons compared to cerebellar Purkinje cells (*p* ≤ 0.003; Online Resource Table 2). The total number of sense RNA foci was significantly greater in frontal cortex neurons than in cerebellar Purkinje cells (*p* = 3.38e-08). Again, findings were similar for antisense RNA foci, except for the total number of foci, which did not reveal a significant difference (*p* = 0.59; Online Resource Table 2).

### Differences between sense and antisense RNA foci

Hereafter, we evaluated differences between sense and antisense RNA foci within cell types. In the frontal cortex, similar patterns were observed, regardless of whether we examined all cells or enriched for neurons. In both instances, the percentage of cells with sense RNA foci was significantly higher than the percentage of cells with antisense RNA foci (*p* ≤ 7.93e-07; Online Resource Table 3). Though there was no significant difference in the mean number of foci overall (*p* ≥ 0.32), in RNA foci-positive cells we did notice that sense RNA foci were less frequently encountered than antisense RNA foci (*p* ≤ 2.50e-07). The maximum number of RNA foci was also significantly smaller for sense compared to antisense RNA foci (*p* ≤ 3.96e-07). Consequently, no significant difference was observed in the total number of RNA foci (*p* ≥ 0.35).

In the cerebellum, the percentage of granule cells with sense RNA foci was significantly higher than that of antisense RNA foci (*p* = 2.52e-08; Online Resource Table 3), but no significant difference was seen in Purkinje cells (*p* = 0.93). This difference was even more profound when examining the mean number of RNA foci in all cells: whereas sense foci were more abundant in granule cells (*p* = 2.52e-08), antisense foci were more frequently encountered in Purkinje cells (*p* = 1.05e-05). Moreover, there was no significant difference in the mean number of RNA foci in positive cells for granule cells (*p* = 0.06), but a significantly greater number was seen for antisense RNA foci in Purkinje cells (*p* = 4.33e-07). Additionally, opposite effects were also observed for the maximum and total number of foci: sense RNA foci were more plentiful in granule cells (*p* ≤ 1.30e-05), and antisense foci in Purkinje cells (*p* ≤ 3.37e-07).

### Significant association with age at onset in frontal cortex

After comparing these groups, we examined whether RNA foci were associated with clinico-pathological features of the disease. Given the fact that correlations were observed between each of the RNA foci measurements (*r* ≥ 0.35, *p* ≤ 0.001; Online Resource Table 4) we focused on one specific measurement: the percentage of cells with RNA foci; however, similar findings were observed for the other measurements.

**Fig. 5:**
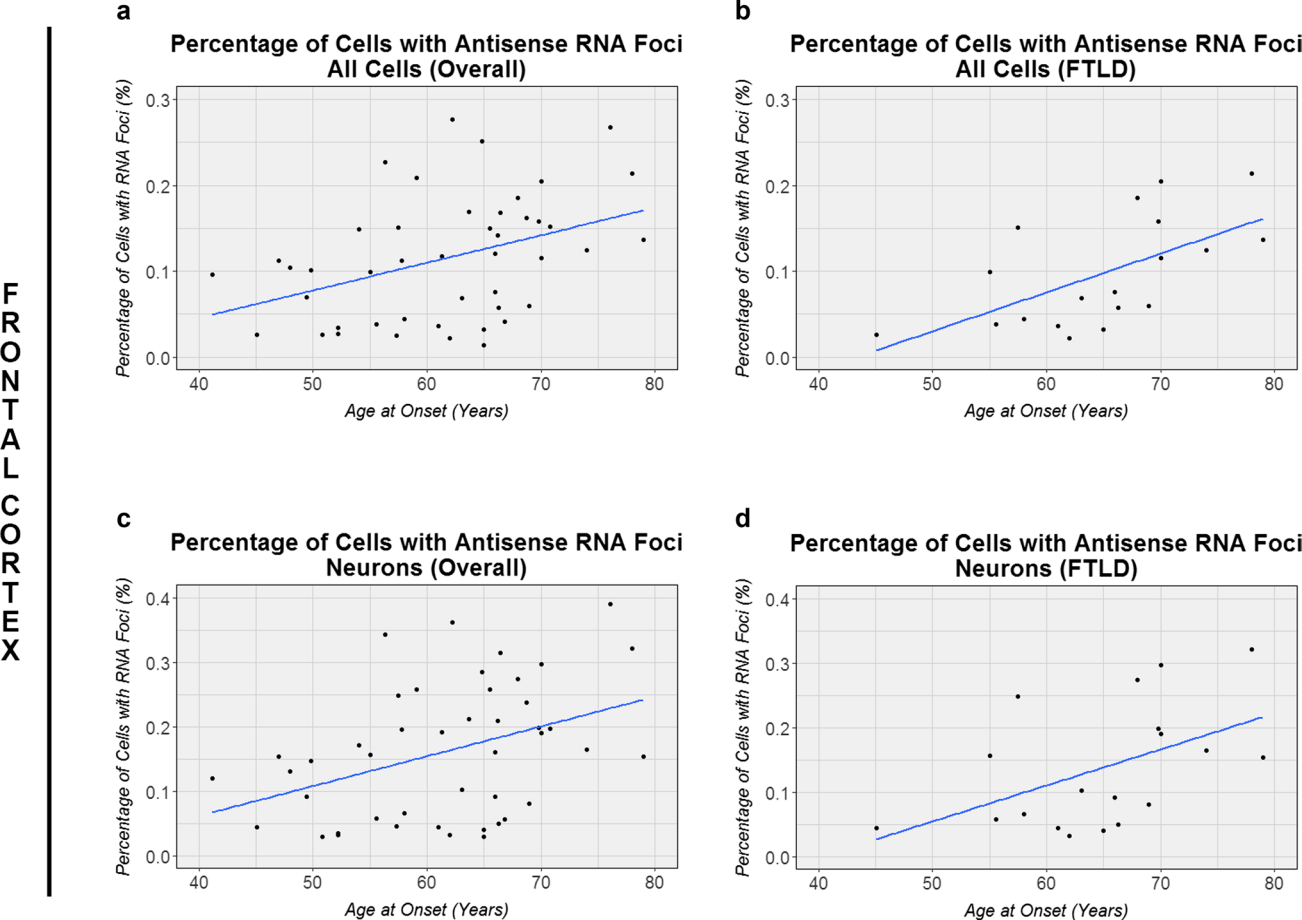
Associations with age at onset. In the frontal cortex, associations with age at onset are shown for our overall cohort of *C9ORF72* expansion carriers, either in all cells (**a**) or in neuronal cells (**c**). Additionally, associations are displayed for the subset of patients diagnosed with frontotemporal lobar degeneration (FTLD), again for all cells (**b**) or when enriching for neurons (**d**). The *solid blue line* denotes the linear regression line, while each individual is represented by a *solid black circle*

We assessed associations with age, repeat length, *C9ORF72* transcript levels, dipeptide-repeat protein levels, gender, disease subgroups, and survival after onset. Interestingly, in our overall cohort of *C9ORF72* expansion carriers, only one significant association was detected that remained significant after adjustment for multiple testing: a higher percentage of cells with antisense RNA foci was associated with a later age at onset in the frontal cortex (*r* = 0.43, *p* = 0.003; Table 3; Fig. 5), which appeared to be most prominent in patients with FTLD (*r* = 0.64, *p* = 0.003; Fig. 5). Findings were comparable when focusing on neurons (*r* = 0.42, *p* = 0.004; Fig. 5).

No significant difference was observed between disease subgroups, nor did we detect significant associations for any of the other variables studied in our overall cohort of *C9ORF72* expansion carriers. Of note, although we focused on our overall cohort to allow assessment of a large number of samples, we included a supplementary table where the results are presented for each of the disease subgroups separately (FTLD, FTLD/MND, and MND; Online Resource Table 5).

**Table 3:** Associations of RNA foci with age, expansion size, *C9ORF72* transcripts, and dipeptide-repeat proteins in two brain regions (overall cohort)

Variable	Association	Spearman’s *r* (95% CI)	*p* value	Spearman’s *r* (95% CI)	*p* value	Spearman’s *r* (95% CI)	*p* value	Spearman’s *r* (95% CI)	*p* value
Frontal cortex		All cells sense		All cells antisense		Neurons sense		Neurons antisense	
Percentage of cells	Age at onset	−0.08 (−0.38 to 0.23)	0.62	0.43 (0.20 to 0.62)	0.003	−0.0008 (−0.31 to 0.30)	1.00	0.42 (0.18 to 0.61)	0.004
	Age at death	−0.16 (−0.43 to 0.13)	0.27	0.27 (−0.007 to 0.51)	0.06	−0.11 (−0.39 to 0.20)	0.47	0.23 (−0.05 to 0.48)	0.11
	Repeat length	−0.19 (−0.52 to 0.16)	0.23	−0.12 (−0.40 to 0.19)	0.46	−0.17 (−0.50 to 0.19)	0.28	−0.17 (−0.46 to 0.15)	0.28
	Total	−0.17 (−0.52 to 0.19)	0.29	−0.19 (−0.47 to 0.10)	0.23	−0.11 (−0.46 to 0.26)	0.52	−0.17 (−0.46 to 0.14)	0.30
	Variant 1	−0.27 (−0.56 to 0.06)	0.10	−0.36 (−0.63 to −0.03)	0.02	−0.23 (−0.53 to 0.10)	0.16	−0.34 (−0.64 to 0.008)	0.03
	Variant 2	0.02 (−0.31 to 0.35)	0.89	−0.13 (−0.44 to 0.22)	0.43	0.05 (−0.29 to 0.37)	0.76	−0.09 (−0.41 to 0.24)	0.57
	Variant 3	0.38 (0.06 to 0.66)	0.02	0.08 (−0.26 to 0.39)	0.60	0.36 (0.03 to 0.64)	0.03	0.09 (−0.26 to 0.42)	0.56
	Intron 1a	−0.26 (−0.54 to 0.09)	0.12	−0.11 (−0.42 to 0.22)	0.50	−0.26 (−0.53 to 0.07)	0.10	−0.14 (−0.44 to 0.19)	0.40
	Intron 1b	0.06 (−0.28 to 0.42)	0.71	−0.13 (−0.49 to 0.22)	0.42	0.008 (−0.32 to 0.36)	0.96	−0.19 (−0.53 to 0.17)	0.25
	Poly(GP)	0.06 (−0.26 to 0.39)	0.72	−0.06 (−0.36 to 0.23)	0.71	−0.02 (−0.31 to 0.29)	0.91	−0.08 (−0.36 to 0.21)	0.63
Cerebellum		Granule cells sense		Granule cells antisense		Purkinje cells sense		Purkinje cells antisense	
Percentage of cells	Age at onset	−0.08 (−0.38 to 0.23)	0.62	−0.26 (−0.54 to 0.05)	0.06	0.02 (−0.25 to 0.29)	0.89	0.23 (−0.05 to 0.46)	0.09
	Age at death	−0.07 (−0.36 to 0.23)	0.62	−0.29 (−0.55 to 0.02)	0.03	0.05 (−0.20 to 0.32)	0.70	0.31 (0.05 to 0.52)	0.02
	Repeat length	−0.09 (−0.41 to 0.26)	0.58	−0.19 (−0.45 to 0.12)	0.18	−0.07 (−0.34 to 0.21)	0.61	0.08 (−0.19 to 0.34)	0.55
	Total	−0.08 (−0.42 to 0.27)	0.61	0.03 (−0.27 to 0.32)	0.81	−0.01 (−0.31 to 0.30)	0.95	−0.22 (−0.47 to 0.07)	0.12
	Variant 1	−0.05 (−0.37 to 0.29)	0.77	0.09 (−0.22 to 0.37)	0.53	−0.09 (−0.38 to 0.22)	0.54	−0.06 (−0.31 to 0.20)	0.67
	Variant 2	−0.30 (−0.62 to 0.03)	0.05	0.21 (−0.08 to 0.47)	0.16	−0.17 (−0.42 to 0.11)	0.26	−0.12 (−0.39 to 0.16)	0.40
	Variant 3	−0.11 (−0.43 to 0.24)	0.50	0.07 (−0.24 to 0.36)	0.65	0.10 (−0.19 to 0.38)	0.50	0.08 (−0.20 to 0.33)	0.59
	Intron 1a	−0.01 (−0.32 to 0.30)	0.93	−0.01 (−0.31 to 0.28)	0.93	0.21 (−0.10 to 0.50)	0.15	−0.10 (−0.40 to 0.20)	0.48
	Intron 1b	0.14 (−0.18 to 0.46)	0.36	0.05 (−0.25 to 0.35)	0.73	0.20 (−0.10 to 0.46)	0.18	−0.002 (−0.29 to 0.28)	0.99
	Poly(GP)	0.01 (−0.34 to 0.35)	0.95	−0.15 (−0.44 to 0.15)	0.29	0.19 (−0.10 to 0.45)	0.19	0.13 (−0.16 to 0.42)	0.34
	Poly(GA)	−0.08 (−0.40 to 0.24)	0.60	0.06 (−0.23 to 0.36)	0.68	0.11 (−0.19 to 0.41)	0.44	0.07 (−0.22 to 0.34)	0.64

Data are Spearman’s correlation coefficient *r* [95% confidence interval (CI)] or *p* value. In the frontal cortex, within our cohort of *C9ORF72* expansion carriers, we examined 13 different associations [age at onset, age at death, repeat length, total *C9ORF72* transcripts, variant 1 transcripts, variant 2 transcripts, variant 3 transcripts, intron 1a containing transcripts, intron 1b containing transcripts, poly(GP) dipeptide-repeat proteins, gender, disease subgroups, and survival after onset] for each outcome, and thus, *p* values below 0.0038 were considered significant after Bonferroni correction; in this table, 10 of those associations are displayed. In the cerebellum, one additional association was examined [poly(GA)], and, therefore, *p* values below 0.0036 were considered significant after Bonferroni correction

### Description of atypical patient pathologically diagnosed with AD

**Fig. 6:**
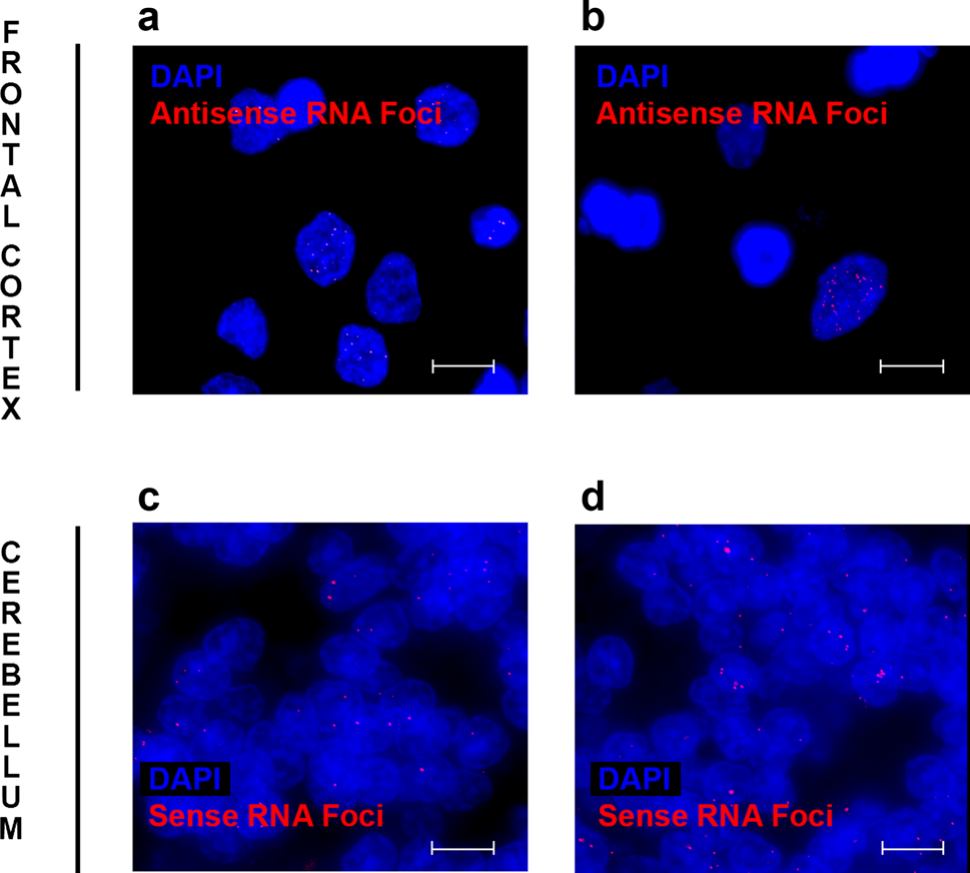
Examples of RNA foci in patient with Alzheimer’s disease (AD). Representative images are shown of a patient with primarily AD pathology who harbored numerous antisense RNA foci in the frontal cortex (**a**, **b**) as well as sense RNA foci in the cerebellum (**c**, **d**). Images contain cell nuclei (*blue* DAPI) and RNA foci (*red* RNA foci). *Scale bars* 5 µm (*top panel*) or 2 µm (*bottom panel*)

**Fig. 7:**
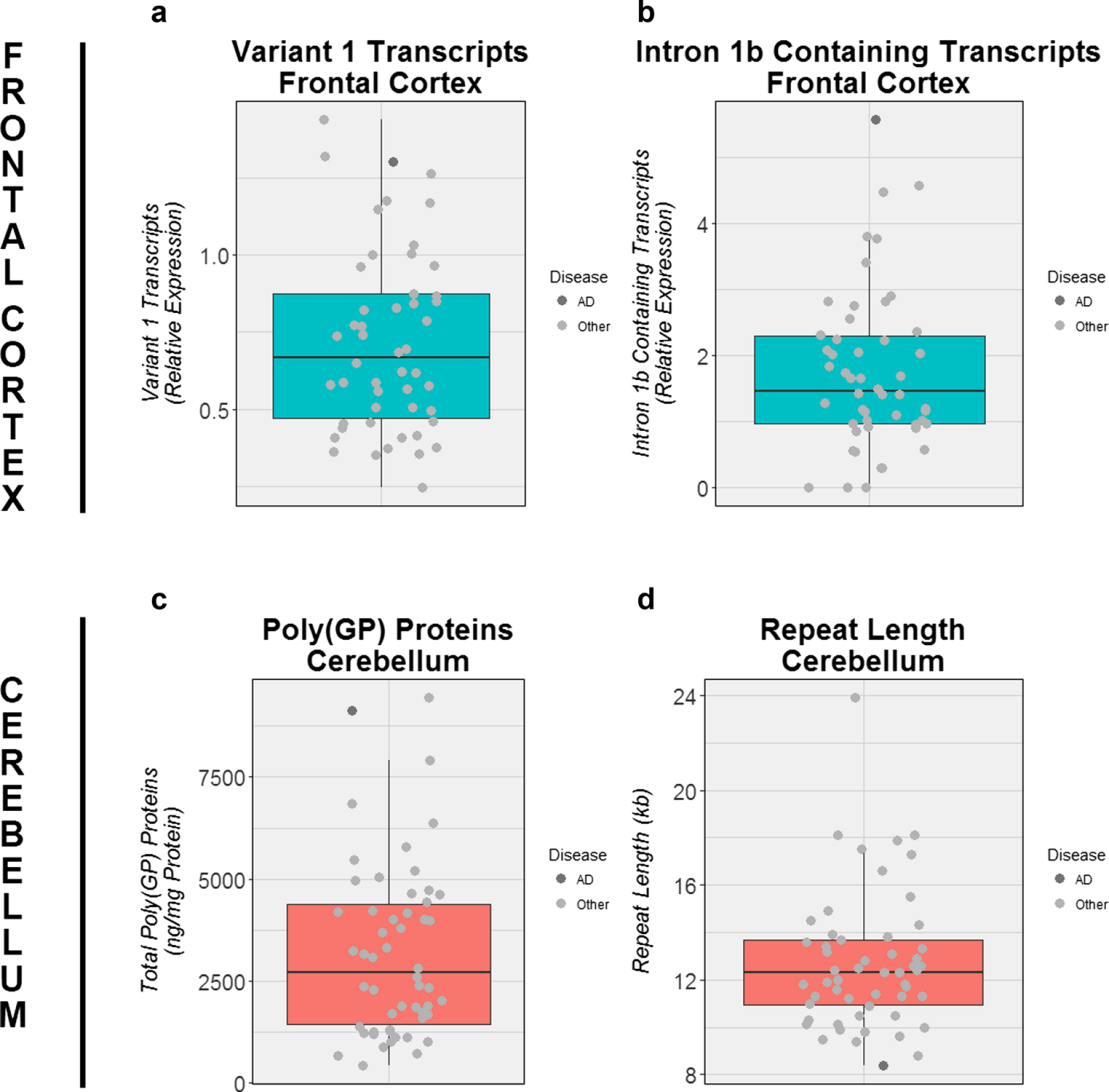
Transcript levels, dipeptide-repeat levels, and repeat length for patient with Alzheimer’s disease (AD). In the frontal cortex (*turquoise boxes*), the expression levels of all *C9ORF72* expansion carriers are shown for *C9ORF72* transcript variant 1 (**a**) as well as for intron 1b containing *C9ORF72* transcripts (**b**). Similarly, poly(GP) dipeptide-repeat protein levels (**c**) and the length of the *C9ORF72* expansion (**d**) are displayed of all *C9ORF72* expansion carriers in the cerebellum (*salmon boxes*). For each *box plot*, the median is represented by a *solid black line*, and each *box* spans the interquartile range (IQR; 25th percentile to 75th percentile). A *solid dark grey circle* is used to denote an expansion carrier with a primary pathological diagnosis of AD; other patients are shown in *light grey*

After performing these analyses, we determined whether any patients existed within our cohort who demonstrated a distinctive RNA foci pattern. We noticed one patient who showed an atypical pattern, and interestingly, this was our only patient with a primary pathological diagnosis of AD [3]. Our patient was a Caucasian woman who began exhibiting memory, language, and psychiatric impairment at 62 years of age, and who was clinically diagnosed with FTD. A brain autopsy was performed when she died at age 65. Macroscopically, diffuse cortical atrophy was appreciable, with pronounced atrophy in the parasagittal regions of the frontal and parietal lobes. Microscopically, the neocortex cortical ribbon was thinned, with neuronal loss and diffuse gliosis. The distribution of tau-immunoreactive neurofibrillary tangles and amyloid-β-immunoreactive senile plaques was consistent with Braak Neurofibrillary Tangle Stage V and Thal Amyloid Phase 5, respectively. Immunohistochemistry for TAR DNA-binding protein 43 (TDP-43) uncovered abundant pathologic lesions in the neocortex; the morphology and distribution of TDP-43-positive inclusions was consistent with TDP-43 harmonized subtype A. Immunohistochemistry for dipeptide-repeat proteins revealed widespread neuronal pathology. Based on these findings, our patient received a diagnosis of primarily AD with secondary FTLD pathology, given the severity of the Alzheimer-type pathology (Online Resource Fig. 8) compared to the TDP-43 pathology with a limited distribution. Remarkably, our AD patient had the highest percentage of antisense RNA foci in the frontal cortex (Figs. 4 and 6; Online Resource Fig. 9): 28% of all cells contained antisense RNA foci and 36% of neurons. Furthermore, she demonstrated the highest percentage of sense RNA foci in the cerebellum (Figs. 4 and 6; Online Resource Fig. 10), with 54% of granule cells harboring sense RNA foci and 89% of Purkinje cells. We subsequently looked at other aspects of *C9ORF72*-related diseases in our AD patient. Importantly, this patient belonged to the most extreme within our group of expansion carriers when examining specific *C9ORF72* transcripts, dipeptide-repeat proteins, and expansion sizes (Fig. 7). More detailed information about this patient is provided in the supplementary information and Online Resource Figs. 8 to 14.

## Discussion

In this study, we performed a thorough characterization of RNA foci in *C9ORF72* expansion carriers, examining sense and antisense RNA foci in two brain regions. Analysis of our entire patient series was possible through our computer-automated pipelines that we optimized to allow recognition of cell nuclei as well as RNA foci. Our detailed analysis revealed differences between cell types, tissue types, and foci types. In the frontal cortex, the percentage of cells with RNA foci was higher in the subset of cells that were designated as neurons than in all cells, while cerebellar Purkinje cells demonstrated a greater percentage of RNA foci than granule cells. When comparing brain regions, the highest RNA foci burden was seen in cerebellar Purkinje cells. In general, sense RNA foci were more common than antisense RNA foci; however, in RNA foci-positive cells antisense foci were usually more abundant than sense RNA foci. We also examined associations with clinical and pathological features of the disease and showed that, in the frontal cortex, a higher percentage of RNA foci appeared to delay age at disease onset, most prominently in FTLD patients. Interestingly, the patient who had the highest RNA foci burden in two brain regions received a primary pathological diagnosis of AD, and also demonstrated exceptional levels of specific *C9ORF72* transcripts, dipeptide-repeat proteins, and expansion sizes.

We are the first to use computer-automated recognition to evaluate RNA foci in *C9ORF72*-linked diseases; however, previous studies used manual counting to examine human brain tissue obtained from a smaller number of individuals. In one study, a detailed characterization of both sense and antisense RNA foci was performed in a cohort of eight *C9ORF72* expansion carriers [26]. Despite differences in sample size and methods, their RNA foci percentages were very similar to those reported in our study: sense RNA foci were detected in 37% of frontal cortex neurons and 21% of cerebellar granule cells, while we observed them in 32 and 23%, respectively. In RNA foci-positive cells, the mean number of sense RNA foci was also comparable: 2.0 versus 2.3 in the frontal cortex and 1.3 versus 1.4 in the cerebellum. These similarities further confirm the validity of our computer-automated pipelines.

In the same study, the authors also examined antisense RNA foci and described that, when antisense RNA foci were present in frontal cortex neurons, they were more common than sense RNA foci. In cerebellar granule cells, they did not observe a similar difference. Again, these findings align with our present study. We would like to emphasize, however, that when we examined the number of RNA foci in foci-positive Purkinje cells, we did observe a significant difference: antisense RNA foci were more abundant. Thus, differences vary based on the type of cells studied.

Other reports have also evaluated RNA foci in *C9ORF72* expansion carriers. One study quantified their numbers in four *C9ORF72* expansion carriers and showed that sense RNA foci were numerous in cerebellar granule cells compared to antisense RNA foci, whereas antisense foci were numerous in cerebellar Purkinje cells [9]. Although this may seem contradictory, these findings are in agreement with our study. Importantly, the authors compared the number of RNA foci in all granule cells or Purkinje cells, not restricting themselves to RNA foci-positive cells. When examining all granule cells, we also noticed that the number of sense RNA foci was greater than that of antisense RNA foci; the opposite pattern was observed in cerebellar Purkinje cells. It is only when we restricted our analysis to cells that harbor RNA foci, that antisense RNA foci were generally more common than sense RNA foci. Furthermore, although not identical, the RNA foci numbers reported in their study were in the same range as those reported in our study, both for granule cells (sense: 0.6 versus 0.3; antisense: 0.02 versus 0.02) and Purkinje cells (sense: 2.6 versus 3.6; antisense: 10.4 versus 7.8). Of note, a third study did not detect significant differences in the percentage of cells with sense or antisense RNA foci in the frontal cortex of four *C9ORF72* expansion carriers [43]; however, their findings were trending and given their relatively small sample size they may not have reached significance. Overall, our comprehensive large-scale study that assessed five different RNA foci measurements in two brain regions of 63 *C9ORF72* expansion carriers highlights similarities between studies, and emphasizes that RNA foci patterns differ depending on the cell type, tissue type, and foci type.

To determine whether the observed RNA foci patterns may result in distinctive (neuronal) injury, we examined associations of RNA foci measurements with age, repeat length, *C9ORF72* transcripts, dipeptide-repeat proteins, gender, disease subgroups, and survival after onset. Remarkably, only a single association was detected that remained significant after adjustment for multiple testing; an increase in antisense RNA foci appeared to delay age at onset in the frontal cortex. This observation may seem counterintuitive: if RNA foci are toxic [20], then one would assume that more RNA foci result in an earlier age at onset. Moreover, since we did observe a similar trend with age at death, we cannot exclude the possibility that RNA foci simply increase when an individual ages and that this might explain the positive association we detected. This would favor a hypothesis in which flawed RNA transcripts accumulate over time, forming RNA foci that may grow in size when they sequester more transcripts and RNA-binding proteins, instead of a hypothesis in which an individual RNA transcript may result in a single RNA focus [22]. It would also be in agreement with reports of RNA foci as neutral intermediates or with neuroprotective effects [37], preventing the export of flawed RNA transcripts that may have given rise to dipeptide-repeat proteins. Thus far, only one other study reported a potential association with age; their analysis of seven *C9ORF72* expansion carriers, indicated that there might be an inverse correlation with the number of RNA foci in the frontal cortex [26]; however, one has to interpret those findings carefully, because of the relatively small number of samples. Additionally, we detected several nominally significant associations in our overall cohort of *C9ORF72* expansion carriers, which did not remain significant after adjustment for multiple testing (Table 3), including associations with specific *C9ORF72* transcripts (e.g., variant 1 and variant 3). Though not discussed in our present manuscript, they may warrant further investigations. Similarly, other studies could examine a larger number of individuals belonging to a particular disease subgroup to evaluate possible associations (Online Resource Table 5).

In previous studies, we already revealed a potential association between specific *C9ORF72* transcripts (variant 1) and survival after onset [39]. Moreover, we revealed that dipeptide-repeat proteins are associated with the cognitive score of clinically diagnosed ALS patients [15]. Examination of their repeat length revealed a possible association with age at onset/collection in the frontal cortex and with survival after onset in the cerebellum [38]. Nevertheless, the majority of the clinical and pathological variability reported in patients carrying a repeat expansion in *C9ORF72* remains unexplained. At the moment, we can only speculate why none of these disease characteristics seems to play a determining role, and postulate that instead of an individual effect of each of these factors it might be their combined effect that is of particular relevance. This seems further substantiated by one of our patients with primarily AD pathology, in whom we identified extreme levels of specific RNA foci, *C9ORF72* transcripts, dipeptide-repeat proteins, and repeat length. We propose that this remarkable combination of findings may have contributed to the atypical presentation in this patient, but we cannot exclude the possibility that the AD pathology was caused by other genetic and/or environmental factors. While we are the first to provide such a detailed description of a *C9ORF72* expansion carrier with primarily AD pathology, other cases have been reported and a repeat expansion in *C9ORF72* is considered to represent a rare cause of AD [3, 5, 16, 18, 41]. Furthermore, our findings have to be interpreted cautiously, since they are based on a single individual.

Although manual counting will remain the gold standard, our computer-automated pipelines provide a reliable estimate of the RNA foci burden. We tried to be conservative, and consequently, our pipelines underestimate the actual number of RNA foci to avoid false-positive findings. Additionally, even though our pipelines appear to recognize specific cell types accurately, they were designed to enrich for those cell types and are not expected to be exclusive. Despite these limitations, our computer-automated pipelines provide an efficient alternative for manual counting, especially for large-scale studies, and it will be interesting to perform a thorough assessment of RNA foci in other neuroanatomical regions (motor cortex, spinal cord, etc.) of *C9ORF72* expansion carriers in future studies. Alternatively, other cerebellar regions could be examined, since our current study concentrated on the Purkinje cell layer that separates the granular and molecular layers (based on reports stating that RNA foci were most often seen in close proximity to this particular layer [14]). Finally, our computer-automated pipelines might be a suitable tool for the evaluation of RNA foci in other repeat expansion disorders, such as myotonic dystrophy and fragile X syndrome.

Though not addressed in our present study, it has previously been shown that RNA foci and dipeptide-repeat proteins infrequently coexist in the same cell [14]. Furthermore, RNA foci can be present in neurons containing p62- or TDP-43-positive inclusions [26]. It has even been suggested that antisense RNA foci correlate with mislocalization of TDP-43 [9], while 77% of motor neurons with antisense RNA foci displayed a loss of nuclear TDP-43 as opposed to 13% of motor neurons without those foci. Regardless of these observations, it is currently unclear how the pieces of the puzzle fit together. Our findings appear to indicate that RNA foci do not represent the sole contributor, which is supported by a report in which a cognitively normal individual with an intermediate number of GGGGCC-repeats (~30 repeats) was described [13]. Intriguingly, although this individual displayed neither symptoms nor TDP-43 pathology, RNA foci and sparse dipeptide-repeat proteins were present in the brain. These findings, therefore, question the pathogenicity of RNA foci and dipeptide-repeat proteins in *C9ORF72*-related diseases, and additionally, they suggest that such a small number of repeats may not trigger the entire disease cascade. A pathological assessment of 35 *C9ORF72* expansion carriers for dipeptide-repeat proteins, also revealed a disconnection between the dipeptide-repeat protein load in inclusion bodies and neurodegeneration [25], whereas they did observe an association between TDP-43 pathology with the phenotype and degeneration. Moreover, other studies reported a disassociation between the distribution of RNA foci and dipeptide-repeat proteins with the regional pattern of neurodegeneration [40]. In line with these findings, we noted that most RNA foci were present in Purkinje cells, even though a loss of Purkinje cells has not been described in the cerebellum of *C9ORF72* expansion carriers [36], indicating that they are spared despite their relatively high RNA foci burden. Furthermore, if the RNA foci burden would correlate with the regional pattern of neurodegeneration, then one would expect to find more RNA foci in the frontal cortex of FTLD patients compared to MND patients, but no significant difference was observed between disease subgroups. As such, it remains unclear which factors drive disease pathogenesis in *C9ORF72*-linked diseases and why only certain regions are affected.

In summary, we conclude from our work that RNA foci are not a strong determining factor in the *C9ORF72* pathomechanism, and postulate a hypothesis in which the combined effect of multiple pathological lesions may lead to the clinical and pathological variability observed in *C9ORF72* expansion carriers.

## Electronic supplementary material

Below is the link to the electronic supplementary material.

Supplementary material 1 (PDF 3661 kb)
