# Supplementary material for: In-depth clinico-pathological examination of RNA foci in a large cohort of C9ORF72 expansion carriers
Source: Acta Neuropathol. 2017 May 15;134(2):255–69. doi: 10.1007/s00401-017-1725-7 (PMC5508036; doi:10.1007/s00401-017-1725-7)
Supplement: Supplementary file 1 — Supplementary material 1 (PDF 3661 kb) [file 401_2017_1725_MOESM1_ESM.pdf]

Original Article Acta Neuropathologica

Electronic Supplementary Material

In-depth clinico-pathological examination of RNA foci in a large cohort of *C9ORF72* expansion carriers

Authors: Mariely DeJesus-Hernandez, NiCole A. Finch, Xue Wang, Tania F. Gendron, Kevin F. Bieniek, Michael G. Heckman, Aliaksei Vasilevich, Melissa E. Murray, Linda Rousseau, Rachael Weesner, Anthony Lucido, Meeia Parsons, Jeannie Chew, Keith A. Josephs, Joseph E. Parisi, David S. Knopman, Ronald C. Petersen, Bradley F. Boeve, Neill R. Graff-Radford, Jan de Boer, Yan W. Asmann, Leonard Petrucelli, Kevin B. Boylan, Dennis W. Dickson, Marka van Blitterswijk, and Rosa Rademakers

Corresponding authors: Marka van Blitterswijk, MD, PhD, Department of Neuroscience, Mayo Clinic, 4500 San Pablo Road, Jacksonville, FL 32224, USA. Telephone number: +1 904 953 2226. Fax number: +1 904 953 7370. Email: VanBlitterswijk.Marka@mayo.edu.

Rosa Rademakers, PhD, Department of Neuroscience, Mayo Clinic, 4500 San Pablo Road, Jacksonville, FL 32224, USA. Telephone number: +1 904 953 6279. Fax number: +1 904 953 7370. Email: Rademakers.Rosa@mayo.edu.

Contents: Description of *C9ORF72* expansion carrier with Alzheimer's disease, Online Resource Table 1–5, and Fig. 1–14

Description of *C9ORF72* expansion carrier with Alzheimer's disease

The patient enrolled in the State of Florida Brain Bank at age 64 and died one year later at age 65. She was a right-handed Caucasian woman with 12 years of education who began exhibiting memory, language, and psychiatric impairment (including paranoid delusions) at 62 years of age. Family history was significant for Alzheimer's disease (AD) in her mother, and unspecified mental illness in her brother. Past medical history was notable for hypertension and total abdominal hysterectomy. She denied alcohol, tobacco, or illicit drug use. She was clinically diagnosed with frontotemporal dementia. Her prescriptions included donepezil hydrochloride (10 mg daily) and quetiapine fumarate (350 mg daily). Magnetic resonance imaging scans at ages 62 and 64 revealed mild diffuse cerebral atrophy and atherosclerotic changes, but were otherwise normal.

The calculated whole brain weight from the formalin-fixed left hemibrain was 840 grams, lower than what would be expected for the individual's age and sex. Macroscopically, diffuse cortical atrophy was appreciable, with more pronounced atrophy in the parasagittal regions of the frontal and parietal lobes, and relatively less atrophy in the temporal lobe. Coronal sections of the brain revealed mild enlargement of the frontal horn and more marked enlargement of the temporal horn. The anterior hippocampus demonstrated severe atrophy whereas the posterior hippocampus and amygdala were mildly atrophic. There was visible neuromelanin pigmentation in the substantia nigra, but loss of pigmentation in the locus coeruleus.

Microscopically, the neocortex cortical ribbon was thinned, with neuronal loss and diffuse gliosis preferentially in the superficial cortical layers. Lewy bodies and neurites were not found in either the amygdala or the substantia nigra. Hematoxylin and eosin as well as thioflavin-S histologic stains revealed many neuritic, cored senile plaques and neurofibrillary tangles in the cortex. Mild leukoariosis and focal amyloid angiopathy (frontal lobe) was noted. Patchy neuronal loss in the hippocampal CA1 and subiculum was associated with many extracelluar neurofibrillary tangles while numerous senile plaques were observed in the pyramidal cell layer and molecular cell layer of the dentate fascia. Other neuroanatomical regions with marked neuronal loss, gliosis, and Alzheimer-type pathology include the entorhinal cortex, the nucleus basalis of Meynert, the amygdala, the hypothalamus, and the basal ganglia. Mild gliosis, neuronal loss, and neurofibrillary tangle pathology were present in the thalamus and substantia nigra while select senile plaques were found in the cerebellar Purkinje and molecular cell layers. The distribution of tau-immunoreactive neurofibrillary tangles and amyloid-β-immunoreactive senile plaques was consistent with Braak Neurofibrillary Tangle Stage V and Thal Amyloid Phase 5, respectively.

Immunohistochemistry using TAR DNA-binding protein 43 (TDP-43) antibodies uncovered abundant pathologic lesions in the neocortex. Neuronal cytoplasmic inclusions, glial cytoplasmic inclusions, neuronal intranuclear inclusions, and short dystrophic neurites, all immunoreactive for TDP-43, were predominantly found in the superficial cortical laminae II. In the hippocampus, TDP-43 pathologic burden was relatively mild compared to the adjacent temporal cortex, and included granular neuronal cytoplasmic inclusions in the dentate fascia and hippocampus proper. The morphology and distribution of TDP-43-positive inclusions was consistent with TDP-43 harmonized subtype A. In addition to the cortical TDP-43 pathology, severe atrophy of the subiculum and fine TDP-43-immunoreactive neurites in the CA1 and subiculum is indicative of concomitant hippocampal sclerosis.

Immunohistochemistry with antibodies against the pooled *C9ORF72* sense dipeptide repeats (Rb5823) revealed widespread neuronal pathology comprising dense neuronal cytoplasmic inclusions, 'star-shaped' neuronal cytoplasmic inclusions, and diffuse cytoplasmic pre-inclusions. In the hippocampus, dipeptide repeat pathology was more marked in the dentate fascia, endplate, CA3, and CA2 compared to TDP-43 pathology (whereas TDP-43 pathology was more pronounced than dipeptide repeat pathology in the CA1 and subiculum). Neuronal intranuclear inclusions and dystrophic neurites composed of dipeptide repeats where rarely observed. Based on these findings, our patient received a diagnosis of primarily AD with secondary frontotemporal lobar degeneration (FTLD) pathology, given the severity of the Alzheimer-type pathology compared to the TDP-43 pathology with a limited distribution.

Remarkably, our AD patient had the highest percentage of antisense RNA foci in the frontal cortex (Fig. 4 and 6; Online Resource Fig. 9): 28% of all cells contained antisense RNA foci and 36% of neurons. Furthermore, she demonstrated the highest percentage of sense RNA foci in the cerebellum (Fig. 4 and 6; Online Resource Fig. 10), with 54% of granule cells harboring sense RNA foci and 89% of Purkinje cells. We then examined eleven additional brain regions, namely the temporal cortex, parietal cortex, motor cortex, visual cortex, hippocampus, cingulate cortex, midbrain, pons, medulla, thalamus, and amygdala. In those regions, we compared our AD patient to a patient with an average number of RNA foci based on observations in the frontal cortex and cerebellum, but we did not detect any obvious differences (Online Resource Fig. 11 and 12). Nevertheless, given our interesting findings in the frontal cortex and cerebellum, we subsequently looked at other aspects of *C9ORF72*-related diseases in our AD patient. Importantly, this patient belonged to the most extreme within our group of expansion carriers when examining specific *C9ORF72* transcripts, dipeptide-repeat proteins, and expansion sizes (Fig. 7; Online Resource

Fig. 13 and 14). In the frontal cortex, for instance, high expression levels of intron containing transcripts as well as variant 1 transcripts were detected, with a relative expression of 806% for intron 1a containing transcripts, 556% for intron 1b containing transcripts, and 130% for variant 1 transcripts. In the cerebellum, relatively high levels of intron 1a containing transcripts (232%) and intron 1b containing transcripts (222%) were also detected, but most profound was the extremely high cerebellar poly(GP) level (amongst the highest observed: 9137 ng/mg protein), whereas the repeat length was smaller than that of other expansion carriers (8.4 kb; ~1000 repeats). Thus, not only did our patient exhibit unusual pathology, an extraordinary combination of features associated with the *C9ORF72* repeat expansion was also noted.

Table 1: RNA foci measurements in frontal cortex and cerebellum comparing cell types (overlap cohort)

Tissue	Variable	Sense			Antisense		
**Frontal Cortex**		All Cells	Neurons	P-value	All Cells	Neurons	P-value
	Percentage of cells	0.28 (0.22–0.31)	0.32 (0.26–0.37)	3.39e-08	0.12 (0.06–0.17)	0.18 (0.09–0.25)	2.52e-08
	Mean number of foci (all)	0.63 (0.46–0.80)	0.80 (0.56–0.97)	2.71e-08	0.51 (0.21–0.87)	0.84 (0.25–1.45)	2.52e-08
	Mean number of foci (pos)	2.20 (1.96–2.52)	2.35 (1.97–2.66)	5.47e-08	4.16 (3.10–5.51)	4.74 (3.43–6.10)	7.24e-06
	Maximum number of foci	11 (9–13)	10 (9–13)	0.31	27 (14–39)	24 (14–39)	0.07
	Total number of foci	213 (152–248)	195 (136–234)	5.37e-08	183 (96–397)	151 (78–311)	5.45e-08
**Cerebellum**		Granule Cells	Purkinje Cells	P-value	Granule Cells	Purkinje Cells	P-value
	Percentage of cells	0.23 (0.16–0.28)	0.71 (0.60–0.80)	2.52e-08	0.02 (0.009–0.03)	0.76 (0.59–0.83)	2.52e-08
	Mean number of foci (all)	0.31 (0.20–0.39)	3.75 (2.88–5.57)	2.52e-08	0.02 (0.01–0.04)	8.52 (4.94–14.79)	2.52e-08
	Mean number of foci (pos)	1.37 (1.28–1.45)	5.35 (3.89–7.57)	2.52e-08	1.32 (1.17–1.44)	13.18 (7.62–16.85)	2.52e-08
	Maximum number of foci	6 (4–7)	15 (10–22)	1.70e-07	4 (3–5)	44 (25–50)	2.46e-08
	Total number of foci	356 (260–479)	43 (30–63)	2.52e-08	44 (20–67)	183 (92–281)	7.53e-08

Data are sample median (IQR) or p-value. In the frontal cortex, RNA foci measurements in all cells are compared to neurons, while RNA foci measurements in granule cells are compared to Purkinje cells in the cerebellum. We performed five similar tests (the percentage of cells with RNA foci, the mean number of RNA foci in all cells, the mean number of RNA foci in foci-positive cells, the maximum number of RNA foci, and the total number of RNA foci), and thus, p-values below 0.01 were considered significant after Bonferroni correction. A paired Wilcoxon signed rank test was used to compare each RNA foci measurement between cell types.

Table 2: RNA foci measurements in frontal cortex and cerebellum comparing tissues (overlap cohort)

**Group**	**Variable**	**Sense**			**Antisense**		
**Neurons versus Granule Cells**		Frontal Cortex	Cerebellum	P-value	Frontal Cortex	Cerebellum	P-value
	Percentage of cells	0.32 (0.26–0.37)	0.23 (0.16–0.28)	3.57e-05	0.18 (0.09–0.25)	0.02 (0.009–0.03)	2.52e-08
	Mean number of foci (all)	0.80 (0.56–0.97)	0.31 (0.20–0.39)	4.05e-07	0.84 (0.25–1.45)	0.02 (0.01–0.04)	2.52e-08
	Mean number of foci (pos)	2.35 (1.97–2.66)	1.37 (1.28–1.45)	2.711e-08	4.74 (3.43–6.10)	1.32 (1.17–1.44)	2.92e-08
	Maximum number of foci	10 (9–13)	6 (4–7)	7.52e-08	24 (14–39)	4 (3–5)	5.60e-08
	Total number of foci	195 (136–234)	356 (260–479)	3.50e-06	151 (78–311)	44 (20–67)	4.09e-06
**Neurons versus Purkinje Cells**		Frontal Cortex	Cerebellum	P-value	Frontal Cortex	Cerebellum	P-value
	Percentage of cells	0.32 (0.26–0.37)	0.71 (0.60–0.80)	2.52e-08	0.18 (0.09–0.25)	0.76 (0.59–0.83)	2.52e-08
	Mean number of foci (all)	0.80 (0.56–0.97)	3.75 (2.88–5.57)	2.52e-08	0.84 (0.25–1.45)	8.52 (4.94–14.79)	2.71e-08
	Mean number of foci (pos)	2.35 (1.97–2.66)	5.35 (3.89–7.57)	4.22e-08	4.74 (3.43–6.10)	13.18 (7.62–16.85)	2.68e-07
	Maximum number of foci	10 (9–13)	15 (10–22)	0.003	24 (14–39)	44 (25–50)	0.002
	Total number of foci	195 (136–234)	43 (30–63)	3.38e-08	151 (78–311)	183 (92–281)	0.59

Data are sample median (IQR) or p-value. RNA foci measurements are shown when comparing neurons in the frontal cortex to granule cells or Purkinje cells in the cerebellum, both for sense and antisense RNA foci. We performed five similar tests (the percentage of cells with RNA foci, the mean number of RNA foci in all cells, the mean number of RNA foci in foci-positive cells, the maximum number of RNA foci, and the total number of RNA foci), and thus, p-values below 0.01 were considered significant after Bonferroni correction. A paired Wilcoxon signed rank test was used to compare each RNA foci measurement between tissue types.

Table 3: RNA foci measurements in frontal cortex and cerebellum comparing RNA foci (overlap cohort)

Tissue	Variable	Sense	Antisense	P-value	Sense	Antisense	P-value
**Frontal Cortex**		All Cells			Neurons		
	Percentage of cells	0.28 (0.22–0.31)	0.12 (0.06–0.17)	1.16e-07	0.32 (0.26–0.37)	0.18 (0.09–0.25)	7.93e-07
	Mean number of foci (all)	0.63 (0.46–0.80)	0.51 (0.21–0.87)	0.52	0.80 (0.56–0.97)	0.84 (0.25–1.45)	0.32
	Mean number of foci (pos)	2.20 (1.96–2.52)	4.16 (3.10–5.51)	1.16e-07	2.35 (1.97–2.66)	4.74 (3.43–6.10)	2.50e-07
	Maximum number of foci	11 (9–13)	27 (14–39)	1.96e-06	10 (9–13)	24 (14–39)	3.96e-06
	Total number of foci	213 (152–248)	183 (96–397)	0.35	195 (136–234)	151 (78–311)	0.86
**Cerebellum**		Granule Cells			Purkinje Cells		
	Percentage of cells	0.23 (0.16–0.28)	0.02 (0.009–0.03)	2.52e-08	0.71 (0.60–0.80)	0.76 (0.59–0.83)	0.93
	Mean number of foci (all)	0.31 (0.20–0.39)	0.02 (0.01–0.04)	2.52e-08	3.75 (2.88–5.57)	8.52 (4.94–14.79)	1.05e-05
	Mean number of foci (pos)	1.37 (1.28–1.45)	1.32 (1.17–1.44)	0.06	5.35 (3.89–7.57)	13.18 (7.62–16.85)	4.33e-07
	Maximum number of foci	6 (4–7)	4 (3–5)	1.30e-05	15 (10–22)	44 (25–50)	3.37e-07
	Total number of foci	356 (260–479)	44 (20–67)	2.52e-08	43 (30–63)	183 (92–281)	9.69e-08

Data are sample median (IQR) or p-value. Sense RNA foci measurements are compared to antisense RNA foci measurements in each of the cell types (frontal cortex: all cells and neurons; cerebellum: granule cells and Purkinje cells). We performed five similar tests (the percentage of cells with RNA foci, the mean number of RNA foci in all cells, the mean number of RNA foci in foci-positive cells, the maximum number of RNA foci, and the total number of RNA foci), and thus, p-values below 0.01 were considered significant after Bonferroni correction. A paired Wilcoxon signed rank test was used to compare each RNA foci measurement between RNA foci types.

Table 4: Associations between RNA foci measurements (overall cohort)

Tissue	Variable	Spearman's *r* (95% CI)	P-value	Spearman's *r* (95% CI)	P-value	Spearman's *r* (95% CI)	P-value	Spearman's *r* (95% CI)	P-value
**Frontal Cortex**		All Cells Sense		All Cells Antisense		Neurons Sense		Neurons Antisense	
Percentage of cells	Number of foci (all)	0.92 (0.83 to 0.96)	<2.2e-16	0.97 (0.92 to 0.99)	<2.2e-16	0.92 (0.82 to 0.97)	<2.2e-16	0.96 (0.91 to 0.97)	<2.2e-16
	Number of foci (pos)	0.63 (0.40 to 0.79)	9.59e-07	0.80 (0.66 to 0.89)	1.91e-12	0.71 (0.50 to 0.84)	1.20e-08	0.77 (0.62 to 0.86)	5.67e-11
	Max number of foci	0.47 (0.21 to 0.67)	0.0006	0.80 (0.63 to 0.89)	4.98e-12	0.50 (0.26 to 0.68)	0.0002	0.77 (0.61 to 0.86)	5.53e-11
	Total number of foci	0.84 (0.66 to 0.94)	8.78e-14	0.95 (0.90 to 0.97)	<2.2e-16	0.82 (0.66 to 0.91)	4.74e-13	0.93 (0.85 to 0.96)	<2.2e-16
**Cerebellum**		Granule Cells Sense		Granule Cells Antisense		Purkinje Cells Sense		Purkinje Cells Antisense	
Percentage of cells	Number of foci (all)	0.99 (0.98 to 1.00)	<2.2e-16	0.97 (0.94 to 0.99)	<2.2e-16	0.70 (0.52 to 0.82)	8.63e-10	0.81 (0.68 to 0.88)	2.28e-15
	Number of foci (pos)	0.78 (0.55 to 0.92)	1.63e-11	0.35 (0.08 to 0.58)	0.007	0.42 (0.16 to 0.64)	0.001	0.65 (0.46 to 0.79)	8.74e-09
	Max number of foci	0.59 (0.33 to 0.78)	5.82e-06	0.53 (0.32 to 0.71)	1.56e-05	0.40 (0.13 to 0.62)	0.002	0.59 (0.37 to 0.74)	5.80e-07
	Total number of foci	0.95 (0.88 to 0.97)	<2.2e-16	0.95 (0.90 to 0.97)	<2.2e-16	0.65 (0.45 to 0.78)	4.62e-08	0.79 (0.66 to 0.87)	1.35e-14

Data are Spearman's correlation coefficient *r* (95% confidence interval [CI]) or p-value. In both brain regions, associations are displayed between the percentage of cells with RNA foci and each of the other RNA foci measurements (number of RNA foci in all cells, number of RNA foci in foci-positive cells, maximum number of RNA foci, and total number of RNA foci). We performed four similar tests (the mean number of RNA foci in all cells, the mean number of RNA foci in foci-positive cells, the maximum number of RNA foci, and the total number of RNA foci), and thus, p-values below 0.0125 were considered significant after Bonferroni correction.

Table 5: Associations of RNA foci with age, expansion size, *C9ORF72* transcripts, and dipeptide-repeat proteins in two brain regions (overall cohort and disease subgroups)

**Tissue**		**C9Plus Cohort**		**FTLD Cohort**		**FTLD/MND Cohort**		**MND Cohort**	
**Frontal cortex (All Cells)**	Association	Spearman's *r* (95% CI)	P-value	Spearman's *r* (95% CI)	P-value	Spearman's *r* (95% CI)	P-value	Spearman's *r* (95% CI)	P-value
Percentage of cells (Sense)	Age at onset	-0.08 (-0.38 to 0.23)	0.62	-0.04 (-0.53 to 0.42)	0.87	0.64 (-0.18 to 0.96)	0.11	-0.29 (-0.74 to 0.27)	0.28
	Age at death	-0.16 (-0.43 to 0.13)	0.27	-0.14 (-0.52 to 0.28)	0.54	0.75 (-0.06 to 1.00)	0.05	-0.27 (-0.74 to 0.29)	0.32
	Repeat length	-0.19 (-0.52 to 0.16)	0.23	-0.15 (-0.66 to 0.35)	0.53	-0.33 (-1.00 to 0.62)	0.44	0.22 (-0.45 to 0.66)	0.49
	Total	-0.17 (-0.52 to 0.19)	0.29	-0.14 (-0.65 to 0.40)	0.57	0.32 (-0.75 to 1.00)	0.47	0.08 (-0.64 to 0.86)	0.80
	Variant 1	-0.27 (-0.56 to 0.06)	0.10	0.07 (-0.43 to 0.57)	0.78	-0.43 (-1.00 to 0.62)	0.37	-0.14 (-0.65 to 0.65)	0.70
	Variant 2	0.02 (-0.31 to 0.35)	0.89	-0.12 (-0.57 to 0.43)	0.64	0.68 (-0.07 to 1.00)	0.09	-0.16 (-0.74 to 0.51)	0.64
	Variant 3	0.38 (0.06 to 0.66)	0.02	0.14 (-0.38 to 0.68)	0.57	0.75 (-0.18 to 1.00)	0.05	0.75 (0.35 to 0.94)	0.007
	Intron 1a	-0.26 (-0.54 to 0.09)	0.12	-0.29 (-0.67 to 0.18)	0.24	-0.36 (-1.00 to 0.65)	0.47	0.34 (-0.27 to 0.82)	0.30
	Intron 1b	0.06 (-0.28 to 0.42)	0.71	0.09 (-0.43 to 0.66)	0.72	0.21 (-0.76 to 1.00)	0.62	0.65 (0.00 to 0.94)	0.03
	Poly(GP)	0.06 (-0.26 to 0.39)	0.72	-0.22 (-0.67 to 0.35)	0.37	-0.64 (-1.00 to 0.18)	0.14	0.24 (-0.50 to 0.73)	0.41
Percentage of cells (Antisense)	Age at onset	0.43 (0.20 to 0.62)	0.003	0.64 (0.26 to 0.86)	0.003	0.43 (-0.33 to 1.00)	0.28	0.53 (0.04 to 0.83)	0.04
	Age at death	0.27 (-0.007 to 0.51)	0.06	0.38 (-0.05 to 0.73)	0.09	0.37 (-0.48 to 0.98)	0.32	0.51 (0.03 to 0.81)	0.04
	Repeat length	-0.12 (-0.40 to 0.19)	0.46	0.34 (-0.08 to 0.63)	0.15	-0.75 (-0.95 to -0.23)	0.01	-0.59 (-0.94 to 0.09)	0.05
	Total	-0.19 (-0.47 to 0.10)	0.23	-0.29 (-0.65 to 0.16)	0.25	-0.13 (-0.82 to 0.81)	0.75	-0.44 (-0.89 to 0.22)	0.19
	Variant 1	-0.36 (-0.63 to -0.03)	0.02	-0.48 (-0.77 to -0.01)	0.05	-0.37 (-0.89 to 0.47)	0.35	-0.55 (-0.89 to 0.20)	0.09
	Variant 2	-0.13 (-0.44 to 0.22)	0.43	-0.30 (-0.68 to 0.20)	0.24	-0.07 (-0.73 to 0.74)	0.88	0.09 (-0.66 to 0.76)	0.78
	Variant 3	0.08 (-0.26 to 0.39)	0.60	-0.02 (-0.52 to 0.47)	0.94	0.25 (-0.65 to 0.88)	0.51	-0.06 (-0.70 to 0.60)	0.86
	Intron 1a	-0.11 (-0.42 to 0.22)	0.50	-0.18 (-0.65 to 0.40)	0.50	0.18 (-0.65 to 0.95)	0.62	-0.50 (-0.89 to 0.20)	0.13
	Intron 1b	-0.13 (-0.49 to 0.22)	0.42	-0.55 (-0.86 to -0.05)	0.02	0.33 (-0.58 to 0.95)	0.37	-0.21 (-0.79 to 0.44)	0.55

	Poly(GP)	-0.06 (-0.36 to 0.23)	0.71	-0.08 (-0.57 to 0.45)	0.76	-0.08 (-0.81 to 0.65)	0.85	-0.28 (-0.75 to 0.32)	0.33
**Frontal cortex (Neurons)**	Association	Spearman's *r* (95% CI)	P-value	Spearman's *r* (95% CI)	P-value	Spearman's *r* (95% CI)	P-value	Spearman's *r* (95% CI)	P-value
Percentage of cells (Sense)	Age at onset	-0.0008 (-0.31 to 0.30)	1.00	0.01 (-0.49 to 0.47)	0.96	0.95 (0.69 to 1.00)	0.0008	-0.13 (-0.57 to 0.43)	0.63
	Age at death	-0.11 (-0.39 to 0.20)	0.47	-0.16 (-0.52 to 0.26)	0.47	0.88 (0.39 to 1.00)	0.008	-0.08 (-0.51 to 0.46)	0.77
	Repeat length	-0.17 (-0.50 to 0.19)	0.28	-0.19 (-0.69 to 0.30)	0.43	-0.19 (-0.90 to 0.75)	0.67	0.22 (-0.47 to 0.76)	0.49
	Total	-0.11 (-0.46 to 0.26)	0.52	-0.11 (-0.62 to 0.44)	0.67	0.50 (-0.56 to 1.00)	0.24	-0.009 (-0.71 to 0.86)	0.99
	Variant 1	-0.23 (-0.53 to 0.10)	0.16	0.04 (-0.46 to 0.55)	0.87	-0.14 (-0.94 to 0.84)	0.79	-0.05 (-0.70 to 0.73)	0.88
	Variant 2	0.05 (-0.29 to 0.37)	0.76	-0.17 (-0.59 to 0.36)	0.49	0.63 (-0.35 to 1.00)	0.12	-0.22 (-0.80 to 0.48)	0.53
	Variant 3	0.36 (0.03 to 0.64)	0.03	0.12 (-0.39 to 0.65)	0.64	0.58 (-0.41 to 1.00)	0.17	0.73 (0.27 to 0.93)	0.01
	Intron 1a	-0.26 (-0.53 to 0.07)	0.10	-0.29 (-0.69 to 0.19)	0.24	-0.40 (-1.00 to 0.56)	0.41	0.36 (-0.25 to 0.82)	0.27
	Intron 1b	0.008 (-0.32 to 0.36)	0.96	0.09 (-0.44 to 0.65)	0.73	-0.07 (-1.00 to 0.80)	0.91	0.63 (-0.009 to 0.91)	0.04
	Poly(GP)	-0.02 (-0.31 to 0.29)	0.91	-0.25 (-0.70 to 0.33)	0.31	-0.50 (-1.00 to 0.43)	0.28	0.03 (-0.65 to 0.60)	0.91
Percentage of cells (Antisense)	Age at onset	0.42 (0.18 to 0.61)	0.004	0.53 (0.11 to 0.80)	0.02	0.76 (0.09 to 1.00)	0.03	0.52 (0.04 to 0.82)	0.04
	Age at death	0.23 (-0.05 to 0.48)	0.11	0.30 (-0.12 to 0.65)	0.19	0.50 (-0.38 to 0.98)	0.17	0.51 (0.04 to 0.81)	0.05
	Repeat length	-0.17 (-0.46 to 0.15)	0.28	0.33 (-0.11 to 0.64)	0.17	-0.82 (-1.00 to -0.37)	0.005	-0.55 (-0.93 to 0.14)	0.07
	Total	-0.17 (-0.46 to 0.14)	0.30	-0.30 (-0.65 to 0.17)	0.25	0.02 (-0.74 to 0.82)	0.95	-0.40 (-0.94 to 0.32)	0.23
	Variant 1	-0.34 (-0.64 to 0.008)	0.03	-0.37 (-0.75 to 0.17)	0.14	-0.37 (-1.00 to 0.51)	0.35	-0.58 (-0.94 to 0.15)	0.06
	Variant 2	-0.09 (-0.41 to 0.24)	0.57	-0.39 (-0.76 to 0.13)	0.12	0.20 (-0.71 to 0.94)	0.59	0.18 (-0.62 to 0.79)	0.59
	Variant 3	0.09 (-0.26 to 0.42)	0.56	0.02 (-0.47 to 0.50)	0.92	0.18 (-0.61 to 0.98)	0.62	-0.08 (-0.72 to 0.63)	0.82
	Intron 1a	-0.14 (-0.44 to 0.19)	0.40	-0.08 (-0.60 to 0.50)	0.76	-0.20 (-0.84 to 0.74)	0.62	-0.51 (-0.90 to 0.22)	0.11
	Intron 1b	-0.19 (-0.53 to 0.17)	0.25	-0.46 (-0.84 to 0.08)	0.06	0.00 (-0.86 to 0.89)	0.98	-0.24 (-0.74 to 0.43)	0.49
	Poly(GP)	-0.08 (-0.36 to 0.21)	0.63	0.02 (-0.46 to 0.52)	0.94	-0.48 (-0.86 to 0.30)	0.20	-0.31 (-0.70 to 0.30)	0.29
**Cerebellum (Granule Cells)**	Association	Spearman's *r* (95% CI)	P-value	Spearman's *r* (95% CI)	P-value	Spearman's *r* (95% CI)	P-value	Spearman's *r* (95% CI)	P-value
Percentage of cells (Sense)	Age at onset	-0.08 (-0.38 to 0.23)	0.62	0.05 (-0.48 to 0.56)	0.85	-0.53 (-0.93 to 0.26)	0.15	-0.10 (-0.59 to 0.41)	0.70
	Age at death	-0.07 (-0.36 to 0.23)	0.62	-0.007 (-0.49 to 0.51)	0.98	-0.32 (-0.90 to 0.54)	0.38	-0.09 (-0.59 to 0.43)	0.75

	Repeat length	-0.09 (-0.41 to 0.26)	0.58	-0.15 (-0.64 to 0.39)	0.58	0.43 (-0.18 to 0.86)	0.19	-0.003 (-0.65 to 0.70)	1.00
	Total	-0.08 (-0.42 to 0.27)	0.61	-0.03 (-0.57 to 0.51)	0.91	-0.25 (-0.80 to 0.57)	0.50	-0.005 (-0.70 to 0.65)	0.99
	Variant 1	-0.05 (-0.37 to 0.29)	0.77	0.05 (-0.50 to 0.56)	0.83	-0.12 (-0.73 to 0.67)	0.77	-0.03 (-0.71 to 0.66)	0.92
	Variant 2	-0.30 (-0.62 to 0.03)	0.05	-0.38 (-0.83 to 0.15)	0.14	-0.24 (-0.93 to 0.54)	0.52	-0.40 (-0.88 to 0.34)	0.19
	Variant 3	-0.11 (-0.43 to 0.24)	0.50	-0.19 (-0.69 to 0.41)	0.47	-0.20 (-0.86 to 0.55)	0.59	0.08 (-0.55 to 0.66)	0.78
	Intron 1a	-0.01 (-0.32 to 0.30)	0.93	0.05 (-0.49 to 0.58)	0.84	-0.42 (-0.82 to 0.23)	0.24	0.15 (-0.37 to 0.63)	0.62
	Intron 1b	0.14 (-0.18 to 0.46)	0.36	0.03 (-0.57 to 0.58)	0.91	0.16 (-0.62 to 0.81)	0.64	0.25 (-0.29 to 0.74)	0.40
	Poly(GP)	0.01 (-0.34 to 0.35)	0.95	-0.08 (-0.58 to 0.44)	0.75	0.13 (-0.54 to 0.77)	0.72	-0.40 (-0.88 to 0.29)	0.14
	Poly(GA)	-0.08 (-0.40 to 0.24)	0.60	0.15 (-0.42 to 0.67)	0.57	-0.13 (-0.84 to 0.60)	0.74	-0.23 (-0.62 to 0.24)	0.41
Percentage of cells (Antisense)	Age at onset	-0.26 (-0.54 to 0.05)	0.06	-0.58 (-0.84 to -0.17)	0.005	-0.12 (-0.71 to 0.56)	0.69	0.14 (-0.35 to 0.64)	0.58
	Age at death	-0.29 (-0.55 to 0.02)	0.03	-0.42 (-0.73 to 0.03)	0.04	-0.10 (-0.69 to 0.60)	0.74	0.17 (-0.32 to 0.65)	0.50
	Repeat length	-0.19 (-0.45 to 0.12)	0.18	-0.12 (-0.61 to 0.44)	0.62	-0.32 (-0.79 to 0.29)	0.27	-0.12 (-0.65 to 0.56)	0.71
	Total	0.03 (-0.27 to 0.32)	0.81	0.09 (-0.38 to 0.52)	0.68	-0.35 (-0.85 to 0.29)	0.25	0.12 (-0.49 to 0.66)	0.70
	Variant 1	0.09 (-0.22 to 0.37)	0.53	0.20 (-0.34 to 0.61)	0.39	-0.34 (-0.86 to 0.35)	0.26	0.36 (-0.26 to 0.77)	0.22
	Variant 2	0.21 (-0.08 to 0.47)	0.16	0.03 (-0.42 to 0.49)	0.90	-0.27 (-0.79 to 0.46)	0.38	0.54 (-0.07 to 0.90)	0.05
	Variant 3	0.07 (-0.24 to 0.36)	0.65	0.02 (-0.46 to 0.50)	0.93	0.24 (-0.43 to 0.74)	0.43	-0.03 (-0.55 to 0.49)	0.92
	Intron 1a	-0.01 (-0.31 to 0.28)	0.93	0.004 (-0.44 to 0.43)	0.98	0.46 (-0.16 to 0.81)	0.11	-0.15 (-0.69 to 0.49)	0.64
	Intron 1b	0.05 (-0.25 to 0.35)	0.73	0.21 (-0.22 to 0.59)	0.35	0.09 (-0.59 to 0.67)	0.76	-0.25 (-0.69 to 0.40)	0.41
	Poly(GP)	-0.15 (-0.44 to 0.15)	0.29	0.13 (-0.34 to 0.56)	0.57	-0.40 (-0.85 to 0.32)	0.18	-0.13 (-0.71 to 0.45)	0.65
	Poly(GA)	0.06 (-0.23 to 0.36)	0.68	0.17 (-0.28 to 0.55)	0.45	-0.20 (-0.78 to 0.48)	0.52	0.36 (-0.24 to 0.75)	0.19
**Cerebellum (Purkinje Cells)**	Association	Spearman's *r* (95% CI)	P-value	Spearman's *r* (95% CI)	P-value	Spearman's *r* (95% CI)	P-value	Spearman's *r* (95% CI)	P-value
Percentage of cells (Sense)	Age at onset	0.02 (-0.25 to 0.29)	0.89	0.38 (-0.006 to 0.68)	0.08	-0.42 (-0.93 to 0.30)	0.21	-0.32 (-0.70 to 0.24)	0.19
	Age at death	0.05 (-0.20 to 0.32)	0.70	0.32 (-0.06 to 0.63)	0.13	-0.23 (-0.82 to 0.51)	0.48	-0.37 (-0.73 to 0.19)	0.13
	Repeat length	-0.07 (-0.34 to 0.21)	0.61	0.09 (-0.34 to 0.52)	0.70	-0.31 (-0.81 to 0.39)	0.31	0.05 (-0.53 to 0.61)	0.87
	Total	-0.01 (-0.31 to 0.30)	0.95	-0.24 (-0.68 to 0.26)	0.32	0.09 (-0.64 to 0.70)	0.77	0.002 (-0.63 to 0.58)	0.99

	Variant 1	-0.09 (-0.38 to 0.22)	0.54	-0.27 (-0.65 to 0.19)	0.25	0.08 (-0.55 to 0.70)	0.79	-0.09 (-0.61 to 0.57)	0.75
	Variant 2	-0.17 (-0.42 to 0.11)	0.26	-0.36 (-0.66 to 0.06)	0.12	-0.03 (-0.65 to 0.55)	0.92	0.11 (-0.47 to 0.72)	0.71
	Variant 3	0.10 (-0.19 to 0.38)	0.50	-0.16 (-0.62 to 0.34)	0.51	0.57 (-0.09 to 0.96)	0.05	-0.13 (-0.72 to 0.48)	0.65
	Intron 1a	0.21 (-0.10 to 0.50)	0.15	0.009 (-0.49 to 0.51)	0.97	0.46 (-0.32 to 0.86)	0.13	0.09 (-0.59 to 0.74)	0.76
	Intron 1b	0.20 (-0.10 to 0.46)	0.18	-0.11 (-0.59 to 0.40)	0.64	0.48 (-0.23 to 0.93)	0.12	0.13 (-0.54 to 0.62)	0.65
	Poly(GP)	0.19 (-0.10 to 0.45)	0.19	-0.26 (-0.63 to 0.18)	0.26	0.74 (0.26 to 0.95)	0.006	-0.13 (-0.58 to 0.35)	0.63
	Poly(GA)	0.11 (-0.19 to 0.41)	0.44	0.05 (-0.47 to 0.57)	0.84	0.43 (-0.26 to 0.81)	0.16	-0.07 (-0.59 to 0.54)	0.80
Percentage of cells (Antisense)	Age at onset	0.23 (-0.05 to 0.46)	0.09	0.08 (-0.40 to 0.48)	0.73	-0.09 (-0.66 to 0.48)	0.77	0.31 (-0.20 to 0.74)	0.21
	Age at death	0.31 (0.05 to 0.52)	0.02	0.15 (-0.32 to 0.53)	0.48	0.02 (-0.53 to 0.57)	0.94	0.36 (-0.16 to 0.77)	0.14
	Repeat length	0.08 (-0.19 to 0.34)	0.55	-0.15 (-0.58 to 0.33)	0.52	0.12 (-0.45 to 0.64)	0.66	0.26 (-0.37 to 0.68)	0.38
	Total	-0.22 (-0.47 to 0.07)	0.12	-0.47 (-0.75 to -0.02)	0.03	-0.59 (-0.84 to -0.15)	0.03	0.21 (-0.36 to 0.72)	0.46
	Variant 1	-0.06 (-0.31 to 0.20)	0.67	-0.06 (-0.45 to 0.35)	0.79	-0.20 (-0.73 to 0.40)	0.50	0.24 (-0.33 to 0.74)	0.41
	Variant 2	-0.12 (-0.39 to 0.16)	0.40	-0.04 (-0.48 to 0.40)	0.87	-0.40 (-0.78 to 0.12)	0.16	0.56 (0.04 to 0.87)	0.04
	Variant 3	0.08 (-0.20 to 0.33)	0.59	-0.26 (-0.65 to 0.20)	0.24	0.04 (-0.53 to 0.52)	0.88	0.24 (-0.34 to 0.73)	0.41
	Intron 1a	-0.10 (-0.40 to 0.20)	0.48	-0.44 (-0.74 to -0.03)	0.04	0.16 (-0.60 to 0.65)	0.58	-0.16 (-0.62 to 0.54)	0.60
	Intron 1b	-0.002 (-0.29 to 0.28)	0.99	-0.36 (-0.70 to 0.09)	0.11	-0.002 (-0.61 to 0.57)	1.00	-0.10 (-0.62 to 0.54)	0.73
	Poly(GP)	0.13 (-0.16 to 0.42)	0.34	-0.10 (-0.60 to 0.39)	0.64	-0.18 (-0.70 to 0.49)	0.54	0.35 (-0.29 to 0.83)	0.19
	Poly(GA)	0.07 (-0.22 to 0.34)	0.64	-0.31 (-0.66 to 0.14)	0.15	0.02 (-0.50 to 0.58)	0.95	0.16 (-0.40 to 0.62)	0.56

Data are Spearman's correlation coefficient *r* (95% confidence interval [CI]) or p-value. In the frontal cortex, within our overall cohort of *C9ORF72* expansion carriers (C9Plus) as well as for each of the disease subgroups (FTLD, FTLD/MND, and MND), we examined 13 different associations (age at onset, age at death, repeat length, total *C9ORF72* transcripts, variant 1 transcripts, variant 2 transcripts, variant 3 transcripts, intron 1a containing transcripts, intron 1b containing transcripts, poly[GP] dipeptide-repeat proteins, gender, disease subgroups, and survival after onset) for each outcome, and thus, p-values below 0.0038 were considered significant after Bonferroni correction; in this table, 10 of those associations are displayed. In the cerebellum, one additional association was examined (poly[GA]), and therefore, p-values below 0.0036 were considered significant after Bonferroni correction. Of note, please interpret these findings carefully, since the sample size is relatively small when restricting the analysis to a specific disease subgroup.

Fig. 1: Different cell types are shown in the frontal cortex

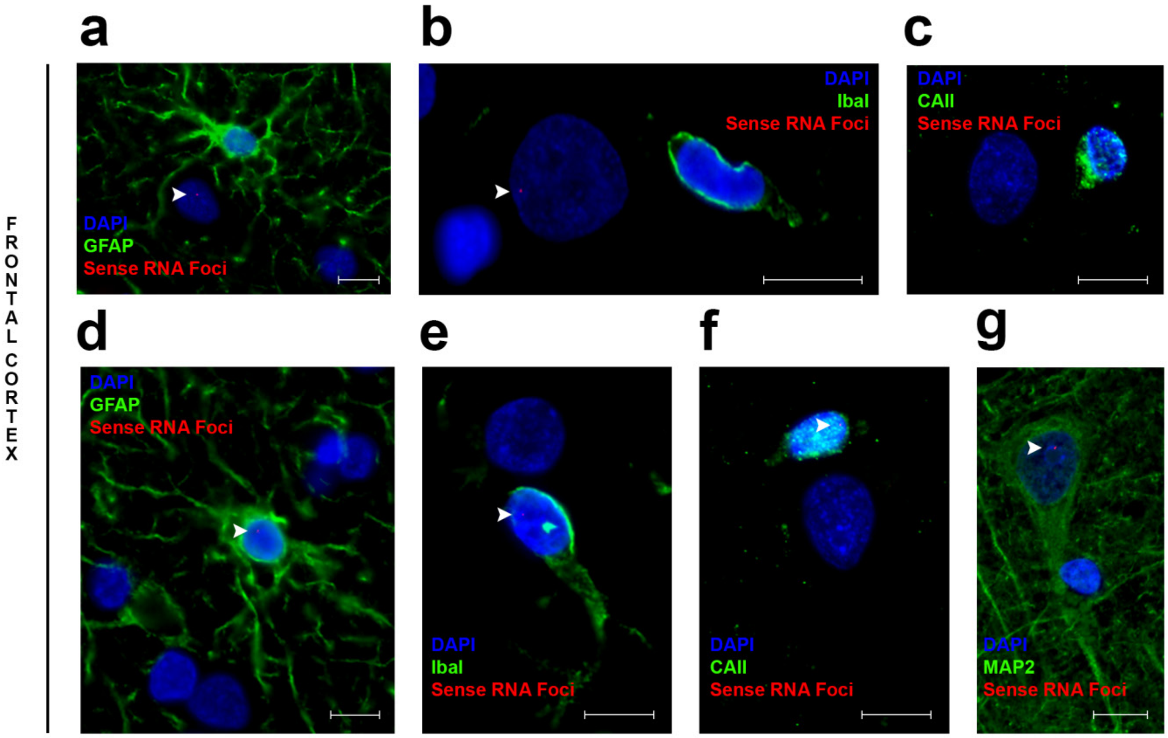

A selection of images is shown to display different cell types in the frontal cortex, including astrocytes (*green* = GFAP; **a**, **d**), microglia (*green* = IbaI; **b**, **e**), oligodendrocytes (*green* = CAII; **c**, **f**), and neurons (*green* = MAP2; **g**). Cell nuclei (*blue* = DAPI) and RNA foci (*red* = RNA foci) are also included. RNA foci that might be difficult to see are highlighted by *arrowheads*. *Scale bar* 5 µm

Fig. 2: Different cell types are shown in the cerebellum

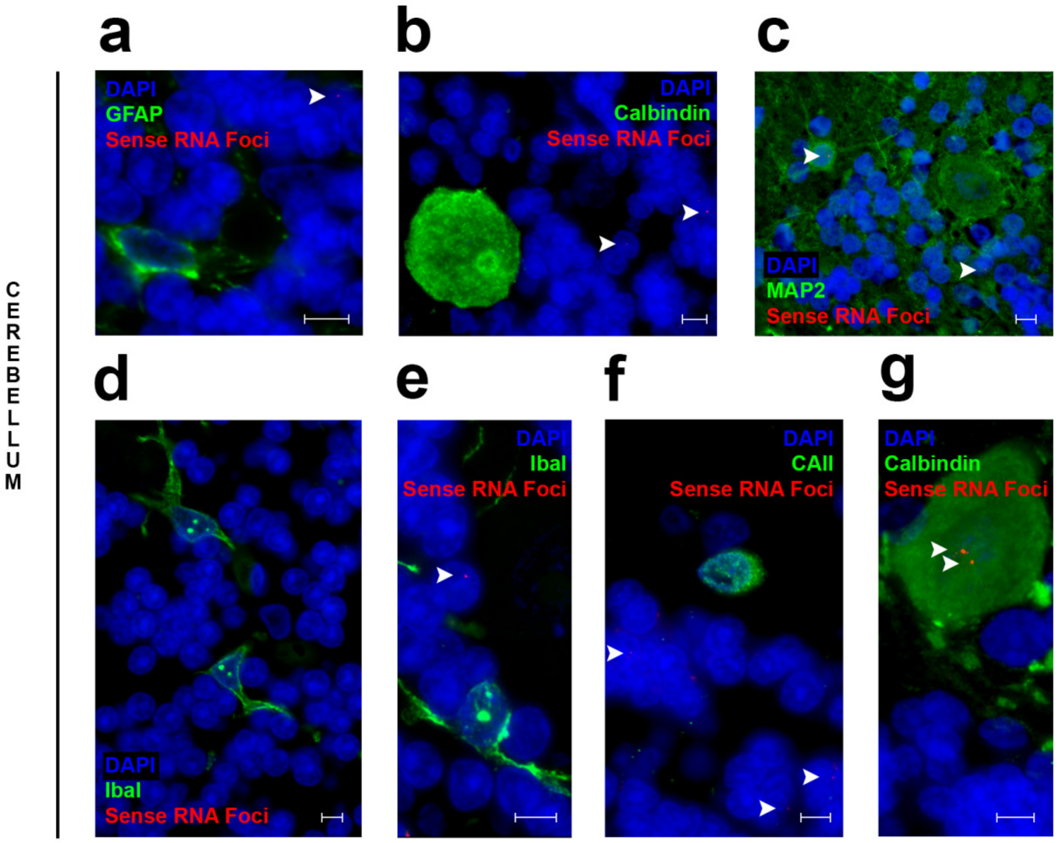

A selection of images is shown to display different cell types in the cerebellum, including astrocytes (*green* = GFAP; **a**), Purkinje cells (*green* = Calbindin; **b**, **g**), neurons (*green* = MAP2; **c**), microglia (*green* = IbaI; **d**, **e**), and oligodendrocytes (*green* = CAII; **f**). Cell nuclei (*blue* = DAPI) and RNA foci (*red* = RNA foci) are also included. RNA foci that might be difficult to see are highlighted by *arrowheads*. *Scale bar* 2 μm

Fig. 3: Examples of computer-automated recognition in two brain regions

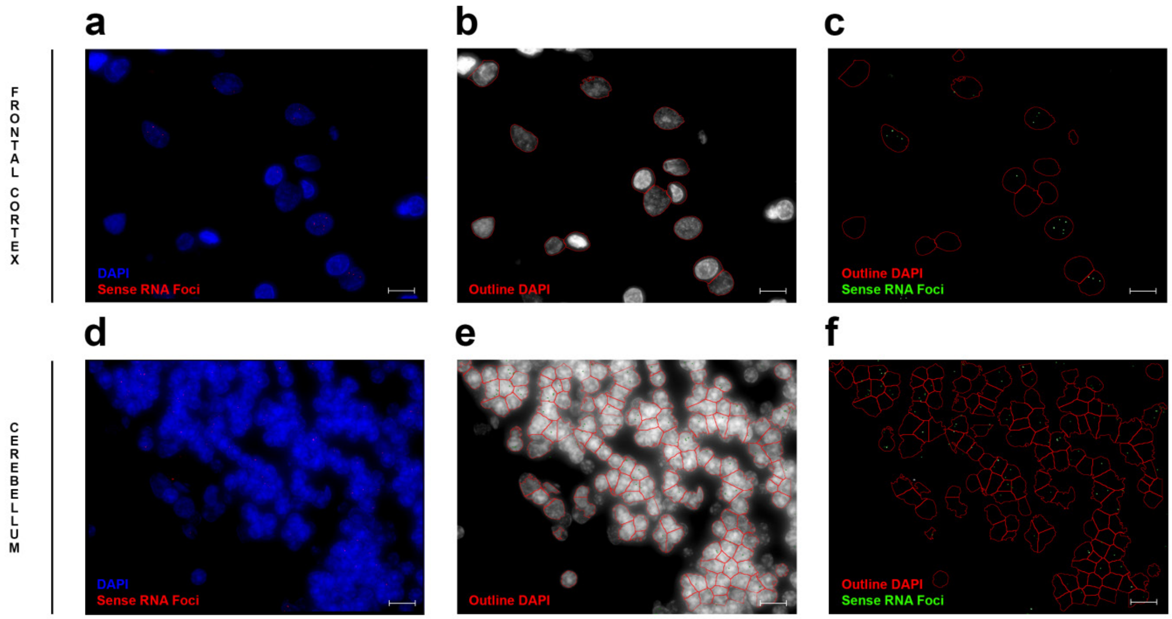

Representative images are displayed of specific steps included in our computer-automated pipelines in the frontal cortex (*top panel*) and cerebellum (*bottom panel*). Merged Z-stack images are shown that contain cell nuclei (*blue* = DAPI) as well as RNA foci (*red* = RNA foci), obtained either in the frontal cortex (**a**) or cerebellum (**d**). Our computer-automated pipelines process these images and recognize individual cell nuclei (*red* = outline nucleus) in the frontal cortex (**b**) or cerebellum (**e**). Subsequently, RNA foci are being recognized (*green* = RNA foci) present in cell nuclei (*red* = outline nucleus), both in the frontal cortex (**c**) and cerebellum (**f**). *Scale bar* 5 µm

Fig. 4: Examples of computer-automated recognition in frontal cortex

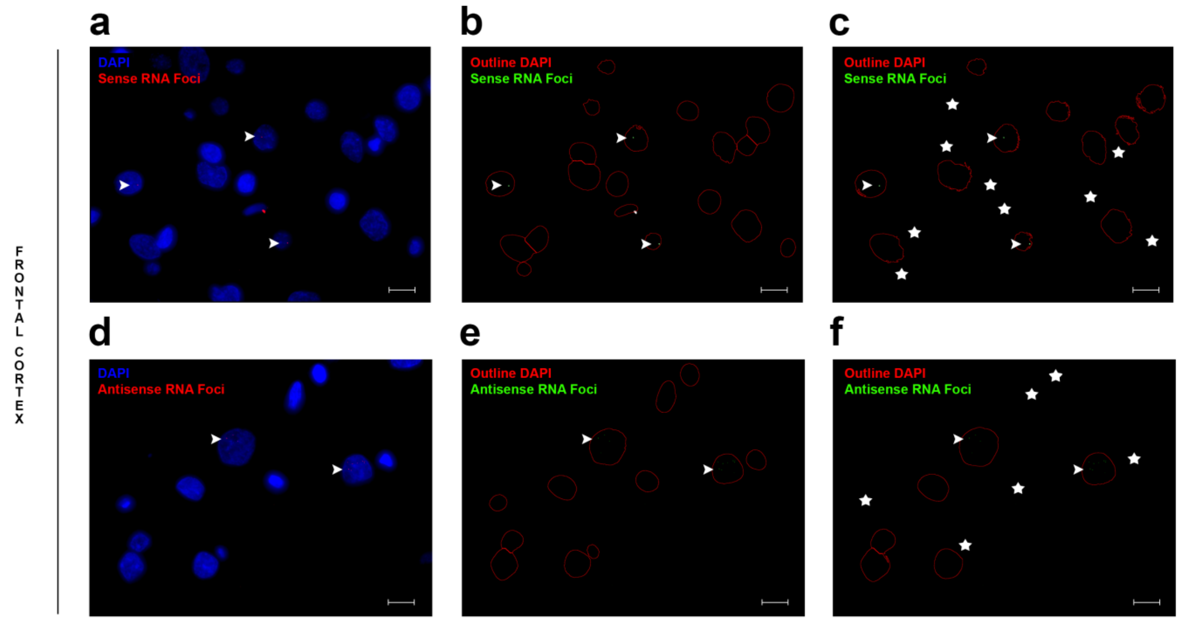

Representative images are shown to explain certain aspects of our computer-automated pipelines in the frontal cortex. Merged Z-stack images contain cell nuclei (*blue* = DAPI) and sense (**a**) or antisense RNA foci (*red* = RNA foci; **d**). Our computer-automated pipelines process these images and recognize either all cell nuclei (*red* = outline nucleus; **b**, **e**) or neuronal cell nuclei (*red* = outline nucleus; **c**, **f**) as well as RNA foci (*green* = RNA foci; **b**, **c**, **e**, **f**). Nuclei that belong to glial cells and that are not included when enriching for neurons are denoted with a *star*. RNA foci that might be difficult to see are highlighted by *arrowheads*. *Scale bar* 5 µm

Fig. 5: Examples of computer-automated recognition in cerebellum

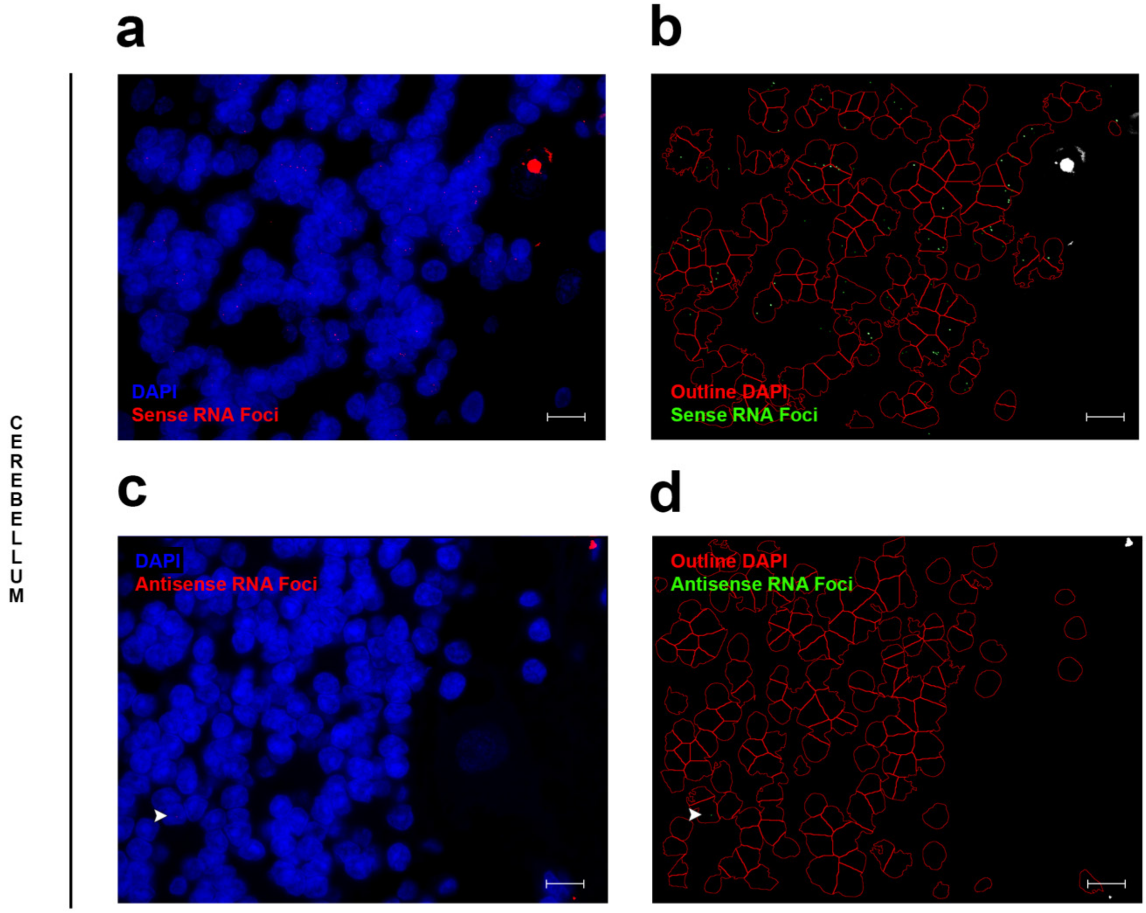

Representative images are shown to explain certain aspects of our computer-automated pipelines in the cerebellum. Merged Z-stack images contain cell nuclei (*blue* = DAPI) and sense (**a**) or antisense RNA foci (*red* = RNA foci; **c**). Our computer-automated pipelines process these images and recognize granule cell nuclei (*red* = outline nucleus; **b**, **d**) as well as RNA foci (*green* = RNA foci; **b**, **d**). RNA foci that might be difficult to see are highlighted by *arrowheads*. *Scale bar* 5 μm

Fig. 6: Validation of computer-automated recognition in frontal cortex

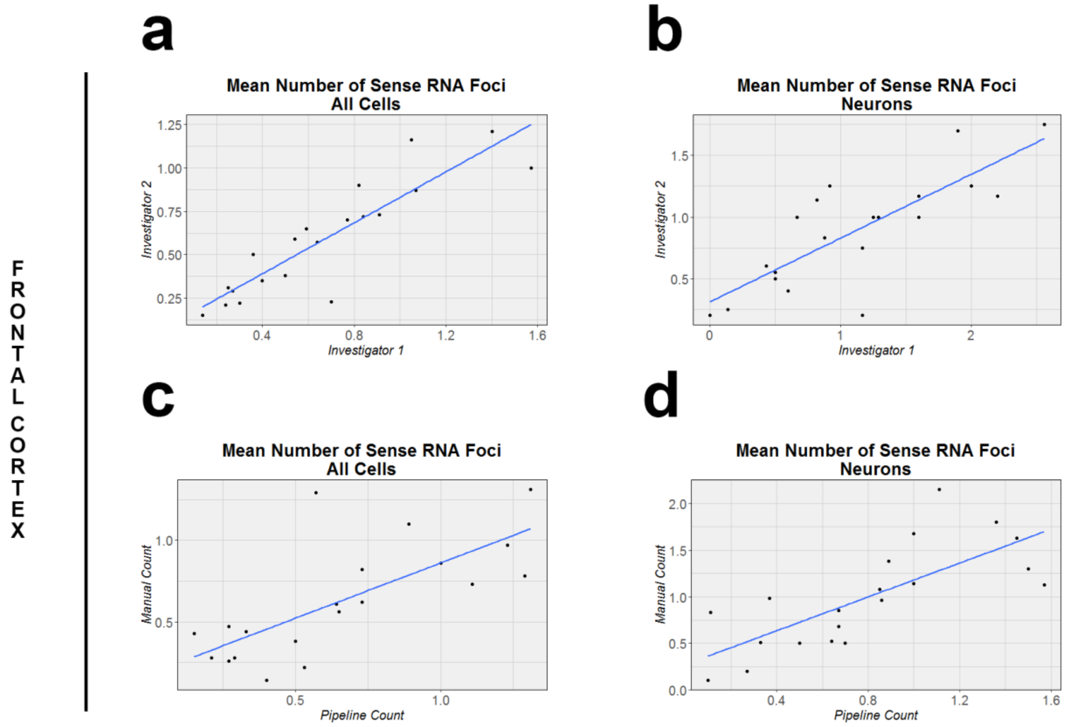

Associations are shown, in the frontal cortex, between two independent investigators who determined the mean number of sense RNA foci in either all cells (**a**) or neurons (**b**), by manual counting. In the same brain region, associations are displayed between our computer-automated pipelines and manual counts for all cells (**c**) as well as for neurons (**d**). The *solid blue line* denotes the linear regression line, while each individual is represented by a *solid black circle*.

Fig. 7: Validation of computer-automated recognition in cerebellum

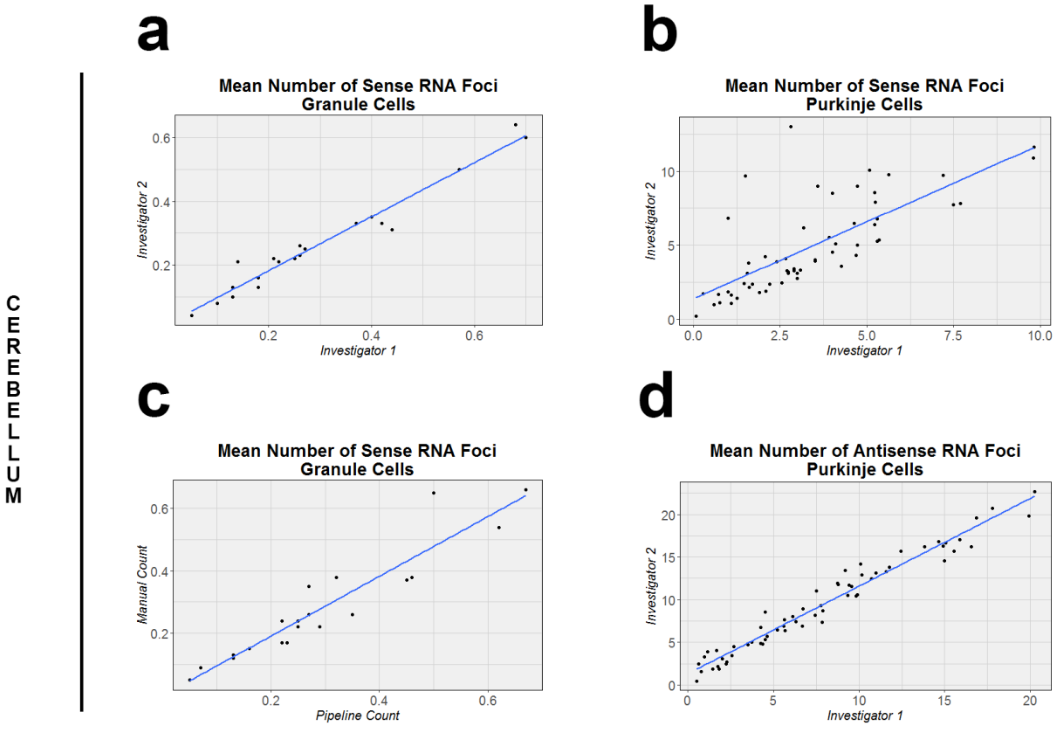

Associations are shown, in the cerebellum, between two independent investigators who determined the mean number of sense RNA foci in either granule cells (**a**) or Purkinje cells (**b**), by manual counting. In the same brain region, associations are displayed between our computer-automated pipelines and manual counting for granule cells (**c**). Since RNA foci were counted manually in Purkinje cells, associations between our pipelines and manual counting could not be examined, but the association between two independent investigators when counting antisense RNA foci is shown instead (**d**). The *solid blue line* denotes the linear regression line, while each individual is represented by a *solid black circle*.

Fig. 8: Alzheimer's disease pathology in a *C9ORF72* repeat expansion carrier

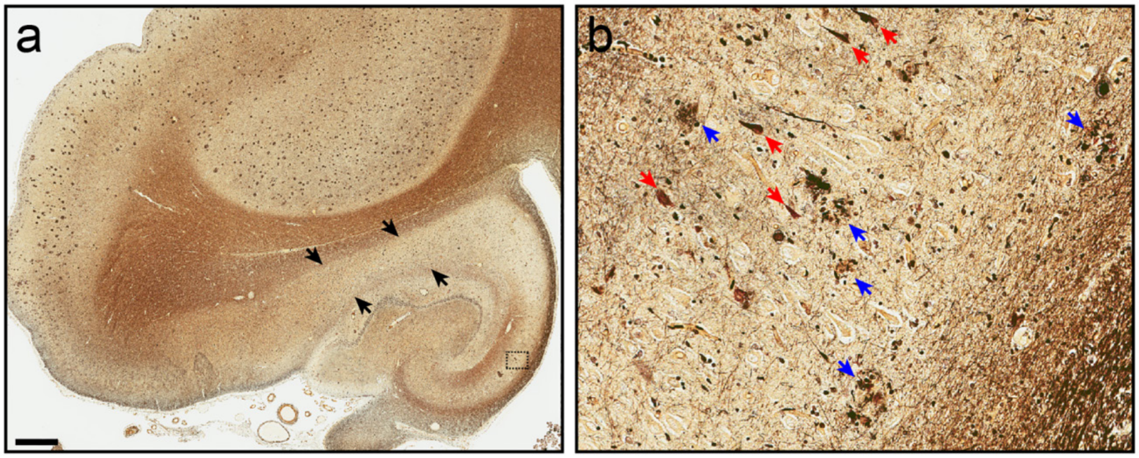

Bielschowsky silver stain of the hippocampus reveals abundant Alzheimer's disease (AD) pathology, including senile plaques and neurofibrillary tangles, in the entorhinal cortex and hippocampus proper. Atrophy of the hippocampus (**a**), especially the subiculum (*black arrows*), is grossly apparent. In the CA2 sector of the hippocampus proper (*inset box*; **b**), neuritic plaques (*blue arrows*) and neurofibrillary tangles (both intracellular and extracellular or "ghost tangles"; *red arrows*) are found in close proximity. *Scale bar* 1 mm (**a**) or 50 µm (**b)**

Fig. 9: Examples of antisense RNA foci in patient with Alzheimer's disease in frontal cortex

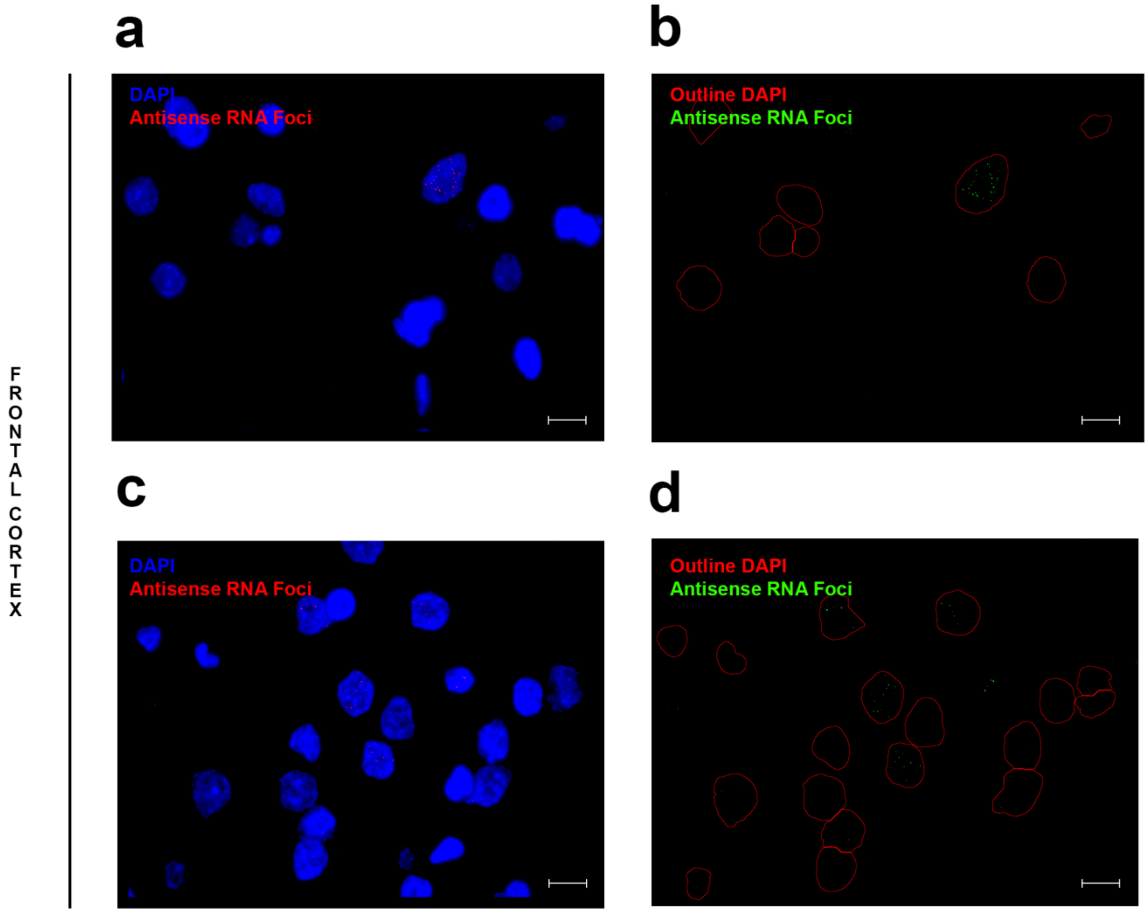

Representative images are shown of a patient with a primary pathological diagnosis of Alzheimer's disease (AD) who harbored numerous antisense RNA foci in the frontal cortex. Merged Z-stack images contain cell nuclei (*blue* = DAPI) and antisense RNA foci (*red* = RNA foci; **a**, **c**). Our computer-automated pipelines process these Z-stack images and recognize neuronal cell nuclei (*red* = outline nucleus; **b**, **d**) as well as RNA foci (*green* = RNA foci; **b**, **d**). *Scale bar* 5 μm

Fig. 10: Examples of sense RNA foci in patient with Alzheimer's disease in cerebellum

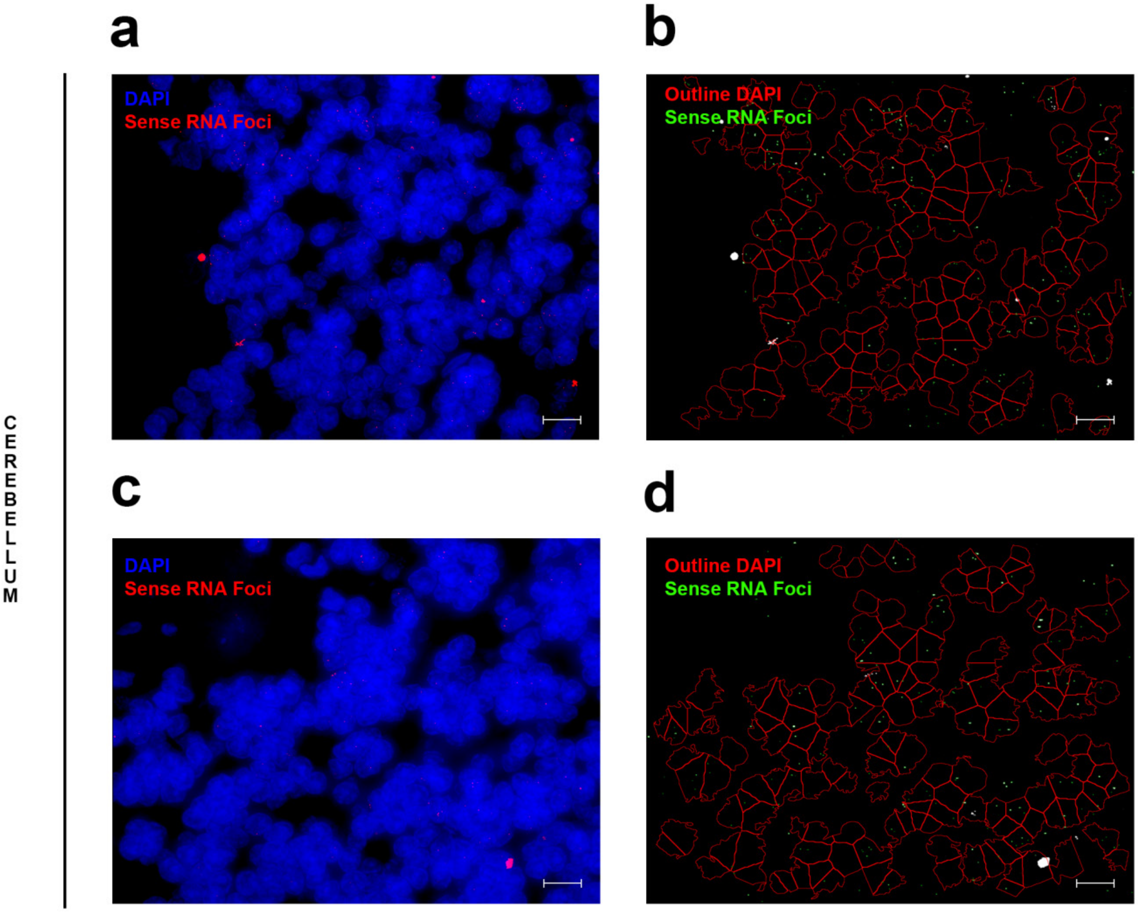

Representative images are shown of a patient with a primary pathological diagnosis of Alzheimer's disease (AD) who harbored numerous sense RNA foci in the cerebellum. Merged Z-stack images contain cell nuclei (*blue* = DAPI) and sense RNA foci (*red* = RNA foci; **a**, **c**). Our computer-automated pipelines process these Z-stack images and recognize granule cell nuclei (*red* = outline nucleus; **b**, **d**) as well as RNA foci (*green* = RNA foci; **b**, **d**). *Scale bar* 5 µm

Fig. 11: Examples of antisense RNA foci in patient with Alzheimer's disease in other brain regions

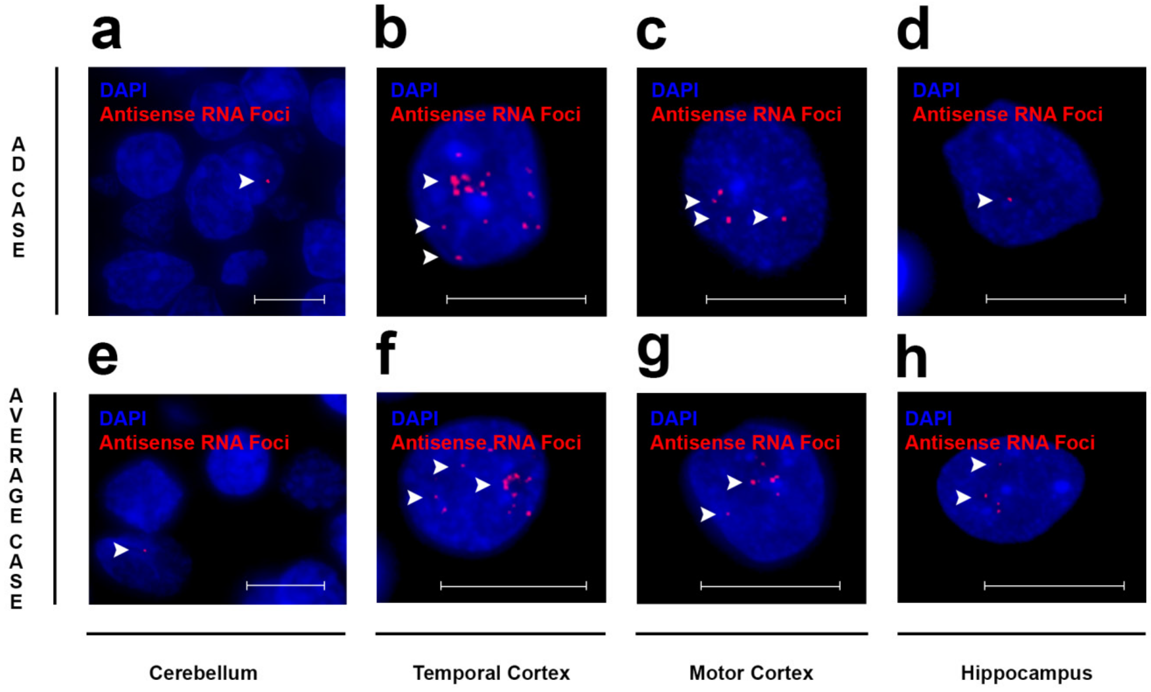

Representative images are shown of antisense RNA foci in a patient with a primary pathological diagnosis of Alzheimer's disease (AD) as well as an average case, examining the cerebellum (**a**, **e**), temporal cortex (**b**, **f**), motor cortex (**c**, **g**), and hippocampus (**d**, **h**). Images contain cell nuclei (*blue* = DAPI) and antisense RNA foci (*red* = RNA foci); no obvious differences are observed between these patients. Of note, other regions are not shown for simplicity. RNA foci that might be difficult to see are highlighted by *arrowheads*. *Scale bars* 5 µm (*temporal cortex*, *motor cortex*, *hippocampus*) or 2 µm (*cerebellum*)

Fig. 12: Examples of sense RNA foci in patient with Alzheimer's disease in other brain regions

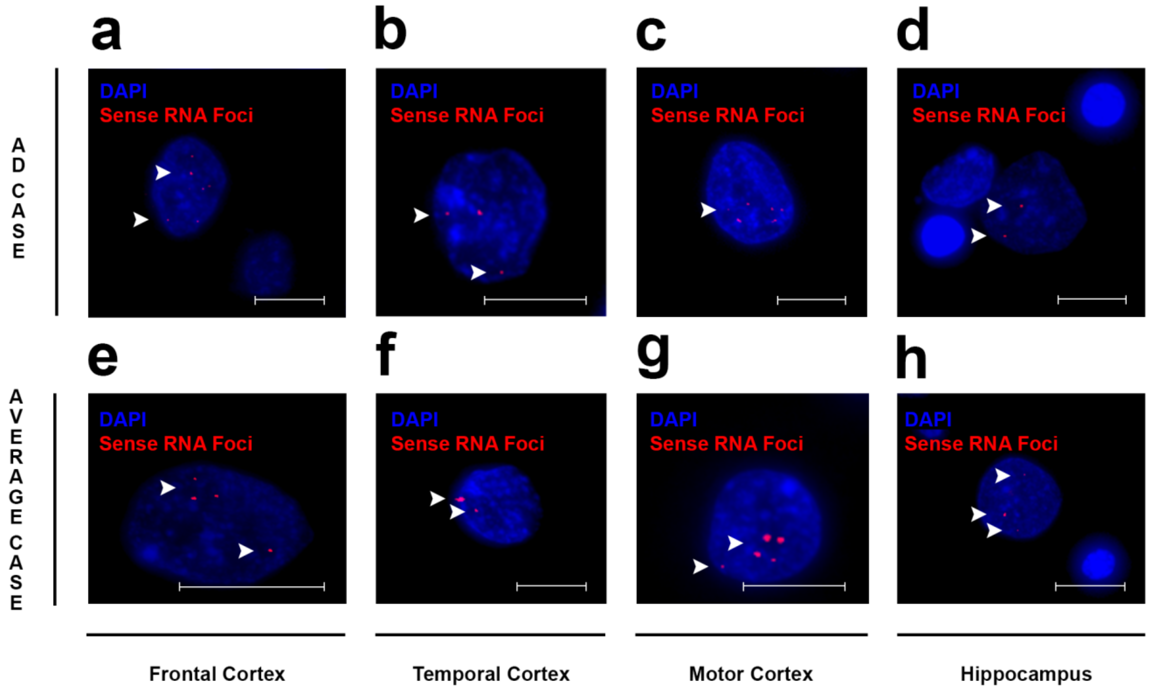

Representative images are shown of sense RNA foci in a patient with a primary pathological diagnosis of Alzheimer's disease (AD) as well as an average case, examining the frontal cortex (**a**, **e**), temporal cortex (**b**, **f**), motor cortex (**c**, **g**), and hippocampus (**d**, **h**). Images contain cell nuclei (*blue* = DAPI) and sense RNA foci (*red* = RNA foci); no obvious differences are observed between these patients. Of note, other regions are not shown for simplicity. RNA foci that might be difficult to see are highlighted by *arrowheads*. *Scale bar* 5 µm

Fig. 13: Transcript levels in the frontal cortex

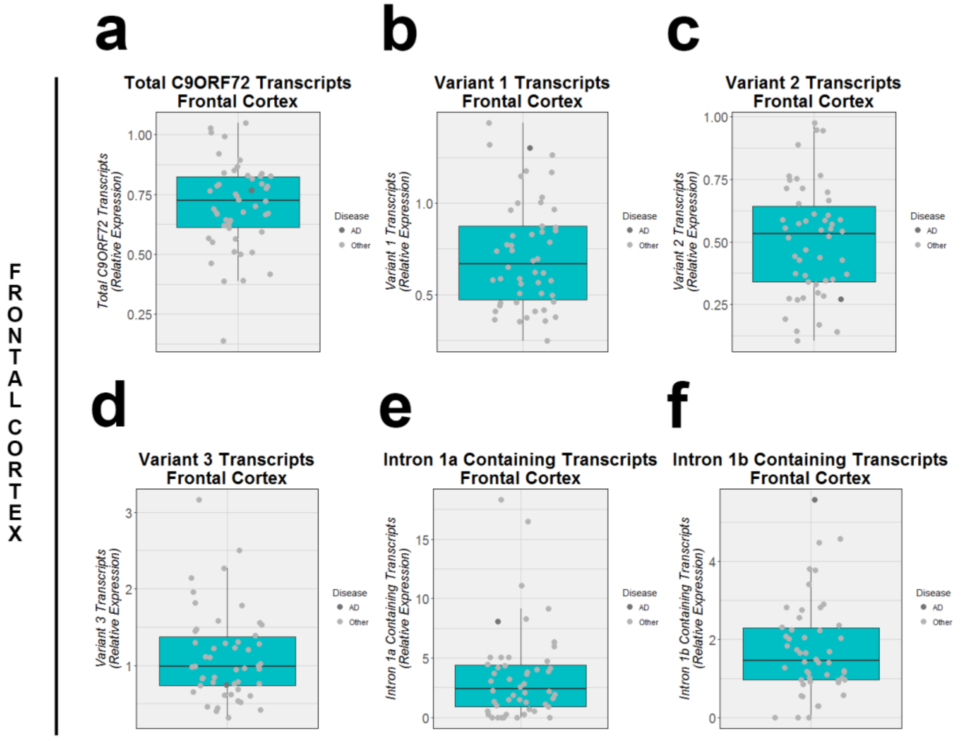

In the frontal cortex (*turquoise* boxes), the expression levels of all *C9ORF72* expansion carriers are shown for total *C9ORF72* transcripts (**a**), *C9ORF72* transcript variant 1 (**b**), *C9ORF72* transcript variant 2 (**c**), *C9ORF72* transcript variant 3 (**d**), intron 1a containing *C9ORF72* transcripts (**e**), and intron 1b containing *C9ORF72* transcripts (**f**). For each box plot, the median is represented by a *solid black line*, and each box spans the interquartile range (IQR; 25th percentile to 75th percentile). A *solid dark grey circle* is used to denote an expansion carrier with a primary pathological diagnosis of Alzheimer's disease (AD); other patients are shown in *light grey*.

Fig. 14: Transcript levels in the cerebellum

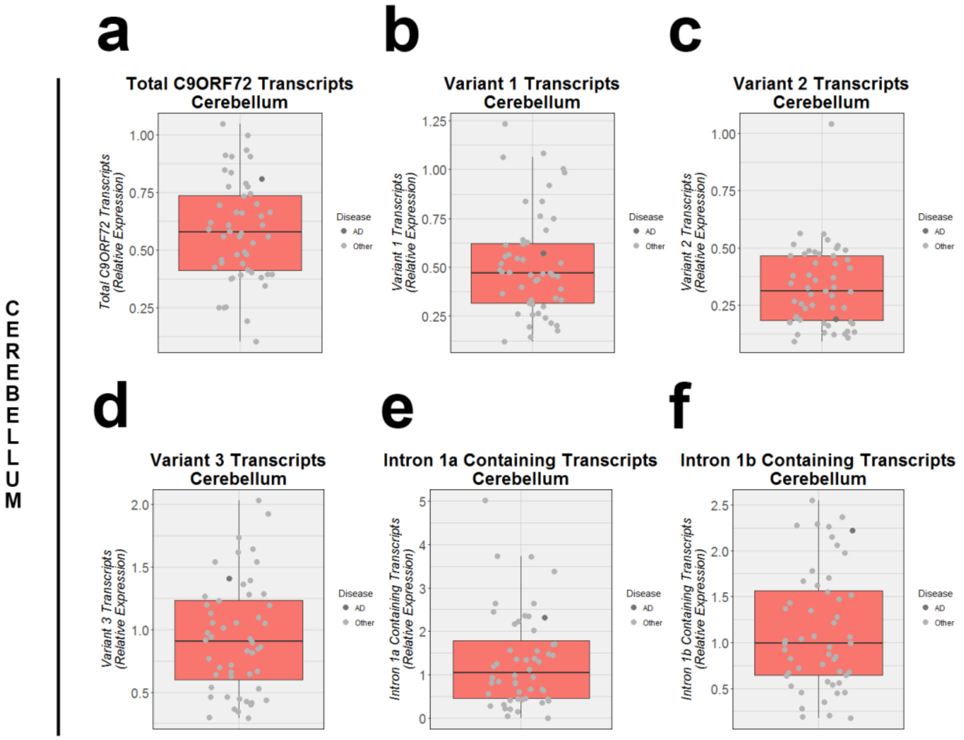

In the cerebellum (*salmon* boxes), the expression levels of all *C9ORF72* expansion carriers are shown for total *C9ORF72* transcripts (**a**), *C9ORF72* transcript variant 1 (**b**), *C9ORF72* transcript variant 2 (**c**), *C9ORF72* transcript variant 3 (**d**), intron 1a containing *C9ORF72* transcripts (**e**), and intron 1b containing *C9ORF72* transcripts (**f**). For each box plot, the median is represented by a *solid black line*, and each box spans the interquartile range (IQR; 25th percentile to 75th percentile). A *solid dark grey circle* is used to denote an expansion carrier with a primary pathological diagnosis of Alzheimer's disease (AD); other patients are shown in *light grey*.
